# Diethyl phthalate (DEP) perturbs nitrogen metabolism in *Saccharomyces cerevisiae*

**DOI:** 10.1038/s41598-022-14284-w

**Published:** 2022-06-17

**Authors:** Corinna Jie Hui Goh, Liang Cui, Jin Huei Wong, Jacqueline Lewis, Megan Goh, Kiat Whye Kong, Lay Kien Yang, Mohammad Alfatah, Yoganathan Kanagasundaram, Shawn Hoon, Prakash Arumugam

**Affiliations:** 1 Bioinformatics Institute, 30 Biopolis Street, Singapore, 138671 Singapore; 2 Antimicrobial Resistance Interdisciplinary Research Group, Singapore-MIT Alliance for Research and Technology, 1 CREATE Way, Singapore, 138602 Singapore; 3 Singapore Institute of Food and Biotechnology Innovation, 61 Biopolis Drive, Singapore, 138673 Singapore; 4 Institute of Molecular and Cellular Biology, 61 Biopolis Drive, Singapore, 138673 Singapore; 5 School of Biological Sciences, Nanyang Technological University, Singapore, 637551 Singapore

**Keywords:** Chemical biology, Genetics, Molecular biology

## Abstract

Phthalates are ubiquitously used as plasticizers in various consumer care products. Diethyl phthalate (DEP), one of the main phthalates, elicits developmental and reproductive toxicities but the underlying mechanisms are not fully understood. Chemogenomic profiling of DEP in *S. cerevisiae* revealed that two transcription factors Stp1 and Dal81 involved in the Ssy1-Ptr5-Ssy5 (SPS) amino acid-sensing pathway provide resistance to DEP. Growth inhibition of yeast cells by DEP was stronger in poor nitrogen medium in comparison to nitrogen-rich medium. Addition of amino acids to nitrogen-poor medium suppressed DEP toxicity. Catabolism of amino acids via the Ehrlich pathway is required for suppressing DEP toxicity. Targeted metabolite analyses showed that DEP treatment alters the amino acid profile of yeast cells. We propose that DEP inhibits the growth of yeast cells by affecting nitrogen metabolism and discuss the implications of our findings on DEP-mediated toxic effects in humans.

## Introduction

Phthalate esters (PAE) are compounds derived by double esterification of phthalic acid (1,2-benzenedicarboxylic acid). Since the Industrial Revolution, low molecular weight phthalates such as dimethyl phthalate (DMP) and diethyl phthalate (DEP) have been used in pharmaceutical and manufacturing industries to confer flexibility to products used in personal care, infant care and medical devices^1^. As PAEs have a propensity to adsorb to soil particles and sediments, they can persist for many years in the environment and bio-accumulate in aquatic organisms and plants^2^. Bioaccumulation factor (BAF) of a phthalate varies inversely as its molecular weight^3^. PAEs can traverse different routes of exposure in humans but the dermal route appears to be most frequent^4^. Several reproductive abnormalities resulting from exposure to phthalates were first observed in rats and were termed as “phthalate syndromes”. Apart from disrupting testosterone production and semen quality^5,6^, DEP has been reported to elicit teratogenic effects as well^7^.

Since phthalates are endocrine-disrupting toxicants^8,9,10,11^ and can readily get leached into the environment^12,3^, regulatory bodies have banned a few phthalates in consumer products. However, the underlying mechanisms of toxicity are not fully understood. Oxidative stress was cited as an important mechanism for phthalate toxicity^13,14^. PAEs were indicated to be uncouplers of oxidative phosphorylation^15,16,17^ whereas other studies stated that they act as inhibitors of electron transport chain and energy transfer^18^. DEP exposure was also reported to result in oxidative stress in onion tissues^19^. DEP caused ultrastructural changes in Leydig cells but its monoester derivative MEP did not^20^. However, extent of human sperm DNA damage positively correlated with DEP’s main urinary metabolite, monoethyl phthalate (MEP)^5,21^. Unlike di-2-ethylhexyl phthalate (DEHP), DEP did not display affinity for estrogen or androgen receptors^22^ and its effect on peroxisomal proliferation was considered to be inconsequential^23,24^.

Identification of genes that regulate resistance or sensitivity to a bioactive compound can help to determine how the bioactive compound affects cellular growth. Chemogenomic profiling studies in *Saccharomyces cerevisiae* reveal the mode-of-action of toxic compounds by pinpointing genes in the yeast genome that regulate sensitivity/resistance to the compound in a single step^25–29^. Our previous studies showed that mono-2-ethylhexyl phthalate (MEHP) perturbs the plasma membrane integrity of *Saccharomyces cerevisiae* cells^25^. In this study, we performed chemogenomic profiling of DEP in *Saccharomyces cerevisiae*. Our studies indicate that DEP interferes with nitrogen metabolism in yeast. Two transcription factors Stp1 and Dal81 involved in the amino acid signalling pathway confer resistance to DEP. Addition of amino acids to the nitrogen-deficient growth medium alleviated DEP toxicity and this rescue was dependent on catabolism of the amino acids via the Ehrlich pathway. Finally, we show that DEP perturbs amino acid homeostasis of yeast cells.

## Results

### DEP but not MEP inhibits the growth of yeast cells

**Figure 1:**
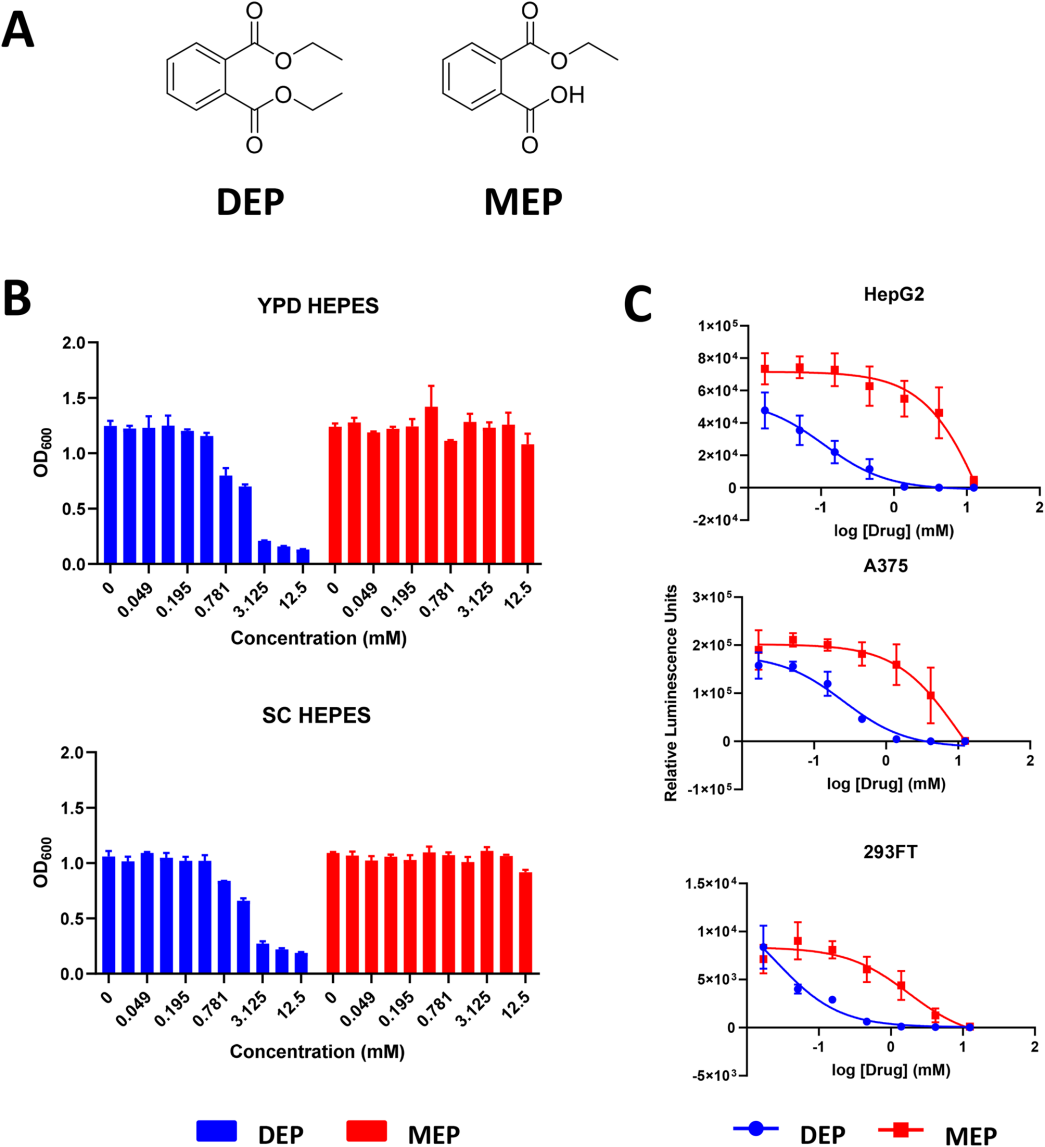
DEP is more toxic than MEP to yeast and human cells. (**A**) Structures of DEP and MEP. (**B**) Growth of wild type yeast cultures (S288C background) in the presence of varying concentrations of MEP and DEP in rich medium (YPD) and Synthetic Complete medium (SC). Each consecutive bar represents a doubled concentration of the preceding bar, with 12.5 mM as the maximal concentration used for both MEP and DEP. (**C**) Plot of relative luminescence units against log(DEP) and log(MEP) from RealTime-Glo MT Cell Viability Assay in HepG2, A375 and 293FT cell lines. Error bars indicate standard deviation (*N* = 2).

To investigate the mode-of-action of DEP (depicted in Fig. 1A) in eukaryotic cells, we explored the possibility of using budding yeast (*Saccharomyces cerevisiae*) as a model. At the outset, we tested whether DEP inhibits the growth of yeast cells. We treated wild type yeast cells (BY4743) with DEP at different concentrations in rich medium (YPD-HEPES) and Synthetic Complete medium (SC) and recorded the growth after 24 h of incubation at 30 °C. DEP completely inhibited the growth of yeast cells at 3.125 mM (Fig. 1B). DEP undergoes hydrolysis to form monoethyl phthalate (MEP)^30^ (Fig. 1A) and has been detected on human skin^31^ and in gastrointestinal tissues^9^. We checked the effect of MEP on the growth of yeast cells. Unlike DEP, MEP did not exert noticeable inhibitory effects on yeast cells in both rich and Synthetic Complete (SC) media (Fig. 1B). This result was surprising as for another phthalate DEHP (di-(2-ethylhexyl)-phthalate), its monoester derivative MEHP (mono(2-ethylhexyl) phthalate) was more toxic in both yeast and mammalian cells^25^. To test if the relative toxicities of DEP and MEP extend to mammalian cells, we compared their effects on the viability of three human cell lines HepG2 (Liver), A375 (Melanoma) and HEK293FT (Human Embryonic Kidney). As observed in yeast cells, DEP was more toxic than MEP in inhibiting the growth of the three human cell lines (Fig. 1C).

### DEP does not affect the membrane integrity of yeast cell

**Figure 2:**
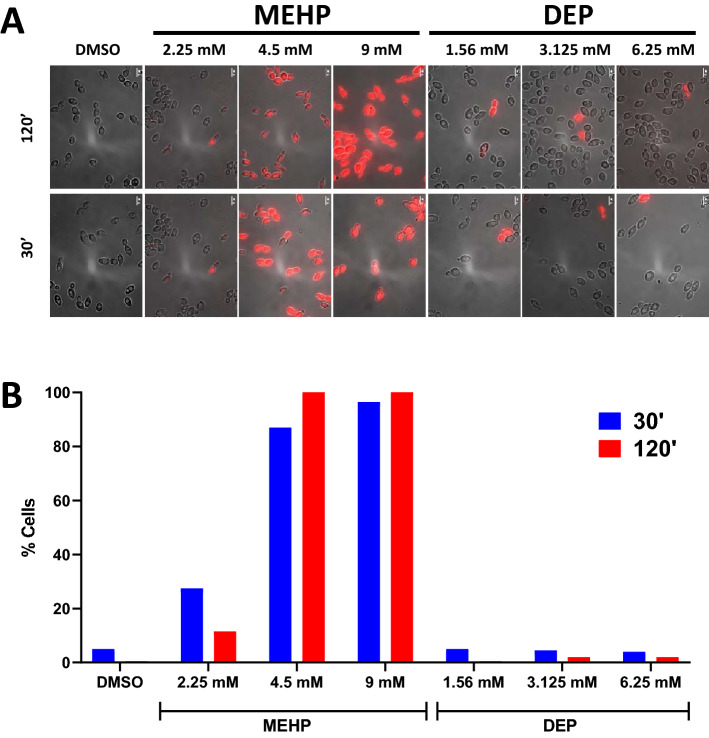
DEP does not affect plasma membrane integrity of yeast cells. (**A**) Representative composite bright field and fluorescence microscopy images of propidium iodide uptake in yeast after 30 min and 2 h exposure to DMSO, MEHP (2.25, 4.5, 9 mM) and DEP (1.56, 3.125, 6.25 mM). Scale bar = 5 µm. (**B**) Percentage of DMSO, MEHP (2.25, 4.5, 9 mM) and DEP (1.56, 3.125, 6.25 mM) treated cells containing propidium iodide (N ≥ 100 cells).

MEHP has been shown to disrupt the membrane integrity of yeast cells^25^. To check if DEP also operates via a similar mechanism, we conducted a membrane integrity assay using the fluorescent dye propidium iodide (PI). PI is a positively charged molecule and cannot enter cells with an intact membrane^32^. We treated logarithmically growing yeast cultures with DEP and MEHP at comparable Inhibitory Concentrations (IC) for 30’ and 120’ and performed the PI-uptake assay. Approximately 97% of yeast (BY4743) cells treated with 4.5 mM MEHP (IC50) internalized PI after 120’, whereas in the same time only 5% of cells treated with 1.56 mM DEP (IC50) contained cytosolic PI staining (Fig. 2A–B). Unlike MEHP, DEP did not induce membrane damage in yeast cells. These results suggest that DEP and MEHP inhibit the growth of yeast cells via distinct mechanisms.

### Chemogenomic profiling of DEP in yeast

**Figure 3:**
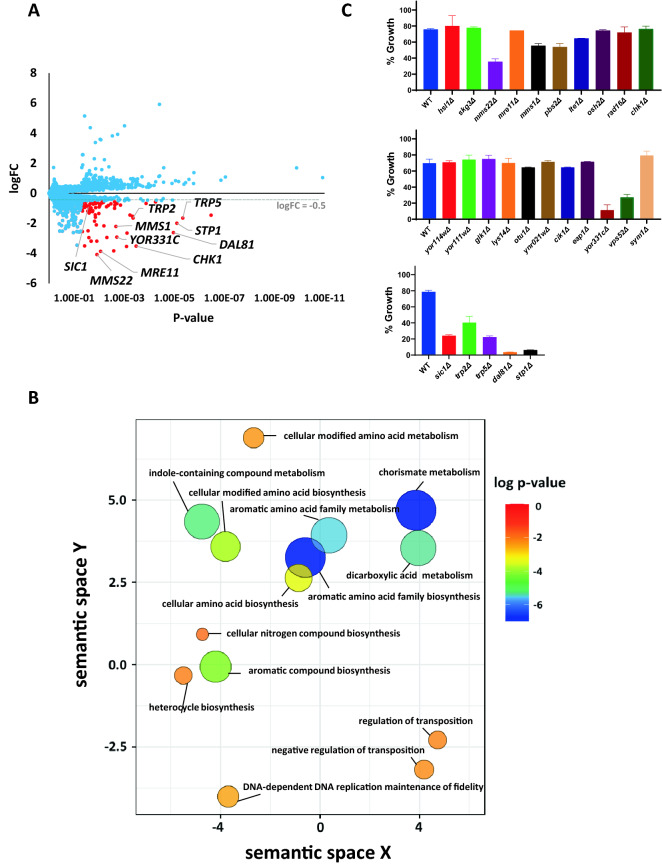
Chemogenomic profiling of DEP. (**A**) Plot of logarithm of fitness coefficient (logFC) versus *P*-value derived from homozygous profiling (HOP) assay of DEP. Mutants with logFC < − 0.5 and *P*-value < 0.05 are displayed as red dots while the rest are displayed as blue dots. Only gene names of the tested mutants deemed significant are annotated in the plot for visualization. The grey dotted line represents logFC of − 0.5. (**B**) Gene ontology (biological process) enrichment analysis of DEP with REVIGO. GO terms obtained from web-based tool DAVID are filtered out by the REVIGO server for redundancy. GO terms which share semantic similarities are clustered together in the 2-dimensional scatterplot. P-value (for the enrichment strength in the annotation category) is depicted by the bubble colour while GO term frequency is depicted by bubble size. (**C**) Validation of HOP data of DEP. Wild type and various deletion mutants were exposed to a series of DEP concentrations in duplicate overnight. Growth measurements (OD_600_) were normalized and plotted for each deletion strain at 1.56 mM DEP. Representative data from one of three biological replicates is shown. Error bars indicate standard deviation (*N* = 2).

As the relative toxicities of DEP and MEP are similar in yeast and human cells, we set out to interpret the mode-of-action of DEP using the chemogenomic profiling approach in yeast. The creation of bar-coded yeast knockout strains from the *Saccharomyces cerevisiae* Genome Deletion Project^33^ enabled the identification of non-essential genes that confer either resistance or sensitivity to a compound^27^^,^^28^^,^^34^. Chemogenomic profiling of the barcoded yeast deletion strains was done at 0.78 mM DEP and the Fitness Co-efficients (FC) of 4817 deletion mutants were computed (Supplementary Table S2). Sensitivity to DEP is indicated by a negative logFC value while resistance to DEP is indicated by a positive logFC value (Fig. 3A). Some of the 4817 mutants had a significantly high absolute logFC value. Mutants with positive logFC values in homozygous profiling assays exhibit virtually no resistance to the compound from our previous work^25,26,35^. Henceforth, we focused on DEP-sensitive mutants (i.e., mutants with logFC value < − 0.5).

To gather insights into DEP’s mode-of-action, Gene Ontology (GO) term enrichment analysis of 78 genes which provide resistance to DEP was done using the online resource tool DAVID^36,37^. We removed the redundant GO terms belonging to different annotation clusters (Supplementary Table S2) with the online server REVIGO^38^. Visualization of GO data by REVIGO (Fig. 3B) indicated that mutants defective in aromatic amino acid metabolism, Nitrogen compound biosynthesis and fidelity of DNA-dependent DNA replication display increased sensitivity to DEP.

To validate the chemogenomic profiling data, we chose 26 mutants *(trp2Δ, hsl1Δ, dal81Δ*, *stp1Δ, sic1Δ, lte1Δ, mms22Δ, mre11Δ, mms1Δ, pbs2Δ, osh2Δ, trp5Δ, yor114wΔ, yor111wΔ, chk1Δ, skg3Δ, rad16Δ, glk1Δ, lys14Δ, eap1Δ, sym1Δ, vps52Δ, cik1Δ*, *yor331cΔ*, *ynr021wΔ* and *otu1Δ*) among the top 78 hits (Supplementary Table S2). Among the 26 mutants tested, eight mutants (30.8% of hits) namely *dal81Δ*, *stp1Δ*, *sic1Δ*, *vps52Δ*, *mms22Δ*, *trp5Δ*, *trp2Δ* and *yor331cΔ* were sensitive to DEP (Fig. 3C) validating the chemogenomic profiling data.

### Mutants defective in DNA repair are not sensitive to DEP

Several DNA-repair defective mutants such as *mms22Δ*, *mms1Δ*, *mre11Δ*, *chk1Δ* and *rtt101Δ* were sensitive to DEP in our chemogenomic profiling assay. Mms22, Mms1 and Rtt1 proteins encode components of the Rtt101^Mms22^ E3-ubiquitin ligase complex which stabilize the replisome at the replication fork^39^. Mre11 is required for repair of DNA double-strand breaks^40^ and Chk1 is a serine-threonine kinase that coordinates the DNA damage response^41^. To examine whether DEP induces genotoxicity, we examined whether mutants defective in DNA damage repair (*rtt107Δ, srs2Δ, rad51Δ, rad27Δ, rad14Δ, rad23Δ, rad4Δ, rad2Δ, psy2Δ* and *pph3Δ*) are also sensitive to DEP. We used a DNA alkylating agent N-Epoxymethyl-1,8-naphthalimide (ENA)^42^ as a positive control. The *rad51Δ* and *mms22Δ* mutants were sensitive to both ENA and DEP (Fig. S1). However, most of the DNA repair defective mutants that were sensitive to ENA were either not sensitive or mildly sensitive to DEP (Fig. S1). These results suggest that DEP does not cause DNA damage.

### *stp1Δ* and *dal81Δ* mutants are sensitive to DEP but not to MEHP

We tested whether a few DEP-sensitive mutants (*dal81Δ*, *stp1Δ*, *sic1Δ*, *mms22Δ* and *trp2Δ*) are also sensitive to MEHP. While *sic1Δ*, *mms22Δ* and *trp2Δ* mutants were sensitive to both MEHP and DEP, the *stp1Δ* and *dal81Δ* mutants were sensitive only to DEP (Fig. S2). Among the various mutants tested, the *stp1Δ* and *dal81Δ* mutants were also most sensitive to DEP (Figs. S2 and 3C). We therefore focused on understanding how Stp1 and Dal81 modulate resistance to DEP.

**Figure 4:**
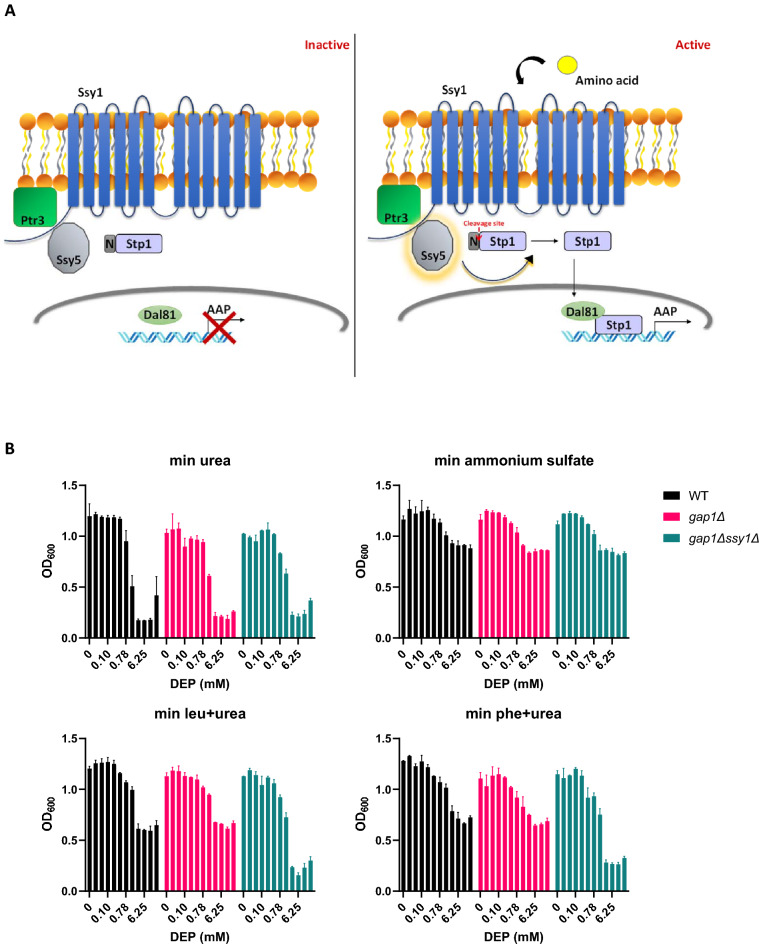
Amino acid addition to a medium containing poor nitrogen source rescues DEP toxicity. (**A**) SPS pathway. When amino acids are absent, SPS pathway remains inactive. Ssy5 remains inactive and does not cleave the pro-domain of Stp1, preventing the transcription of amino acid permease (AAP) genes. When amino acids are present, SPS pathway is active. Upon binding to the amino acid sensor Ssy1, Ssy5 is activated and cleaves the pro-domain of Stp1, permitting it to enter the nucleus and associate with Dal81 to induce transcription of amino acid permease (AAP) genes. (**B**) Growth inhibition of WT, *gap1Δ* and *gap1Δ ssy1Δ* (Σ1278b background) in minimal urea media with no amino acids or with 5 mM ammonium sulfate or phenylalanine, or leucine. Error bars indicate standard deviation (*N* = 2). Each consecutive bar represents a doubled concentration of the preceding bar, with 25 mM as the maximal concentration used for DEP.

*DAL81* and *STP1* encode transcription factors that form a part of the Ssy1p-Ptr3p-Ssy5p (SPS) signalling system (Fig. 4A) deployed by yeast cells in response to extracellular amino acids^43^. This pathway couples the transcription of amino acid permease (AAP) genes to the presence of extracellular amino acids. The amino acid permease homolog Ssy1 functions as an amino acid receptor as a part of the plasma membrane-localized Ssy5-Ptr3-Ssy1 (SPS) sensor complex. In the absence of extracellular amino acids, Stp1 is retained in the cytosol via its inhibitory N-terminal domain. Amino acid binding to SPS sensor complex activates the protease Ssy5 which cleaves off the inhibitory N-terminal domain of transcription factor Stp1. Following cleavage, Stp1ΔN relocates to the nucleus and in collaboration with Dal81 induces expression of amino acid permease genes such as *AGP1*, *BAP2*, *BAP3*, *DIP5, GAP1*, *GNP1*, *TAT1*, *TAT2* and *PTR2*^44,45^.

### DEP toxicity is enhanced in growth medium containing poor nitrogen source

We hypothesized that import of exogenous amino acids into yeast cells might alleviate the toxic effects of DEP. To test the effect of amino acid addition on DEP’s toxicity, yeast cells should be able to grow in the absence of extracellular amino acids. We therefore used an amino acid prototrophic strain Σ1278b for our experiments. We first tested whether the quality of nitrogen source affects the sensitivity to DEP. We compared the effect of DEP on yeast cells grown in medium containing ammonium sulfate (a preferred nitrogen-rich source) and urea (a poor nitrogen source)^46^. 3.125 mM DEP completely inhibited the growth of wild type, *gap1Δ* and *gap1Δ ssy1Δ* yeast cells in minimal medium with urea as a nitrogen source (Fig. 4B, upper left panel). In contrast, 12.5 mM DEP had only a slight effect on the growth of these yeast cells in minimal medium with ammonium sulfate as a nitrogen source (Fig. 4B, upper right panel). These results are consistent with the hypothesis that DEP interferes with nitrogen metabolism in yeast.

### Addition of amino acids to poor nitrogen medium rescues DEP toxicity

To check if addition of amino acids to the urea-containing medium rescues DEP toxicity, we exposed wild type cells to different concentrations of DEP in minimal urea medium with or without 5 mM phenylalanine/5 mM leucine and checked the growth after 48 h of incubation at 30 °C. Interestingly, addition of either phenylalanine or leucine to the minimal urea medium greatly reduced DEP-toxicity in wild-type cells (Fig. 4B, lower panels). Uptake of amino acids from the extracellular medium is mediated by the Gap1 (general amino acid permease) and amino acid permeases like Agp1, Bap2 and Bap3 that are regulated by the SPS-signalling pathway. We therefore tested whether the amino-acid mediated rescue of the DEP-toxicity is dependent on Gap1 and the SPS pathway. Addition of amino acids rescued DEP toxicity in wild type and *gap1Δ* cells (Fig. 4B, lower panels). However, amino acid addition failed to rescue the toxicity of DEP in *gap1Δ ssy1Δ* cells (Fig. 4B, lower panels). These results suggest that transport of amino acids mediated by Gap1 and SPS-signalling pathway is required for the rescue of DEP-induced toxicity in minimal urea medium.

To determine if the rescue of toxicity by amino acid addition could be applicable to MEHP as well, we tested the effect of amino acid addition on the toxicity of MEHP. Addition of phenylalanine (2.5 mM/5 mM/10 mM Phe) to the medium rescued DEP toxicity but was unable to rescue MEHP toxicity (Fig. S3). This is consistent with earlier observations that MEHP but not DEP perturbs plasma membrane integrity^25^ (Fig. 2) and that *stp1Δ* / *dal81Δ* mutants are not sensitive to MEHP (Fig. S2).

### DEP does not inhibit the SPS amino acid sensing pathway

**Figure 5:**
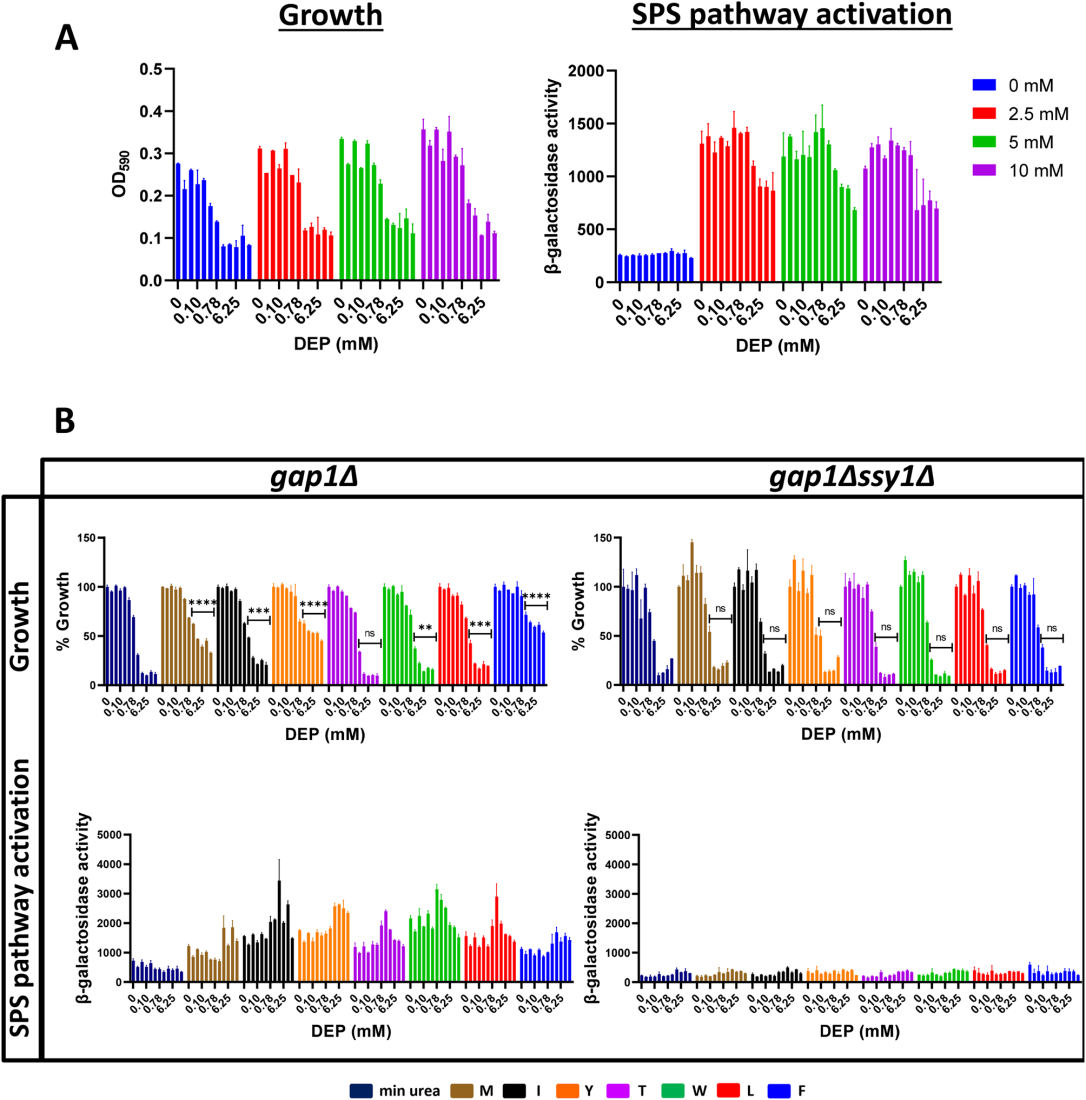
DEP does not affect the activation of the SPS signalling pathway. (**A**) OD_600_ and β-galactosidase activity in *gap1Δ* (Σ1278b background) with DEP at different phenylalanine concentrations (0, 2.5, 5 and 10 mM). Error bars indicate standard deviation (*N* = 2). Two-fold serial dilution of DEP (maximum concentration at 25 mM) was used. (**B**) Comparison of normalized growth and β-galactosidase activity in *gap1Δ* and *gap1Δ ssy1Δ* (Σ1278b background) with and without individual amino acids (5 mM) methionine (M), isoleucine (I), tyrosine (Y), threonine (T), tryptophan (W), leucine (L) and phenylalanine (F) at a series of DEP concentrations. Error bars indicate standard deviation (*N* = 2). Two-fold serial dilution of DEP (maximum concentration at 25 mM) was used. (One-tailed paired *t*-test: ****P ≤ 0.0001, ***P ≤ 0.001, **P ≤ 0.01, *P ≤ 0.05 and ns: *P* > 0.05).

As DEP toxicity can be rescued by the addition of amino acids, we probed whether DEP inhibits the SPS-sensing pathway. To measure the activation of the SPS-signalling pathway, we used the *gap1*Δ strain that expresses the lacZ reporter gene from high-Affinity Glutamine Permease 1 (*AGP1*) promoter. We treated yeast cells with either DMSO or DEP in the presence/absence of phenylalanine and measured the growth (OD_600_) and promoter activity (lacZ activity) after 48 h of incubation at 30 °C. As expected, addition of amino acids was required for activating *AGP1*-lacZ expression in wild type cells (Fig. 5A). Although DEP inhibited the growth of yeast cells, it did not affect *AGP1* promoter activity (Fig. 5B), suggesting that DEP does not inhibit the activation of SPS-pathway.

### Different amino acids rescue DEP toxicity to variable extents

We then compared the effect of adding various amino acids on rescue of DEP-toxicity and induction of the SPS-signalling pathway. As expected, addition of amino acids activated *AGP1*-lacZ expression in *gap1Δ* cells but not in *gap1Δ ssy1Δ* cells (Fig. 5B). In the absence of amino acids in the growth medium, *AGP1-lacZ* activity remained relatively low in wild type cells (Fig. 5B). All the amino acids used in this study induced the *SPS-*signalling pathway (Fig. 5B) but differed in their ability to suppress the toxic effects of DEP. Barring threonine, all the other amino acids tested were able to alleviate DEP toxicity. Notably, the aromatic amino acids phenylalanine and tyrosine were the most effective rescuers of DEP toxicity. Tryptophan, on the other hand, had a modest effect on rescuing DEP toxicity although it strongly induced the *AGP1* promoter.

### Activation of SPS pathway is insufficient to confer resistance to DEP

**Figure 6:**
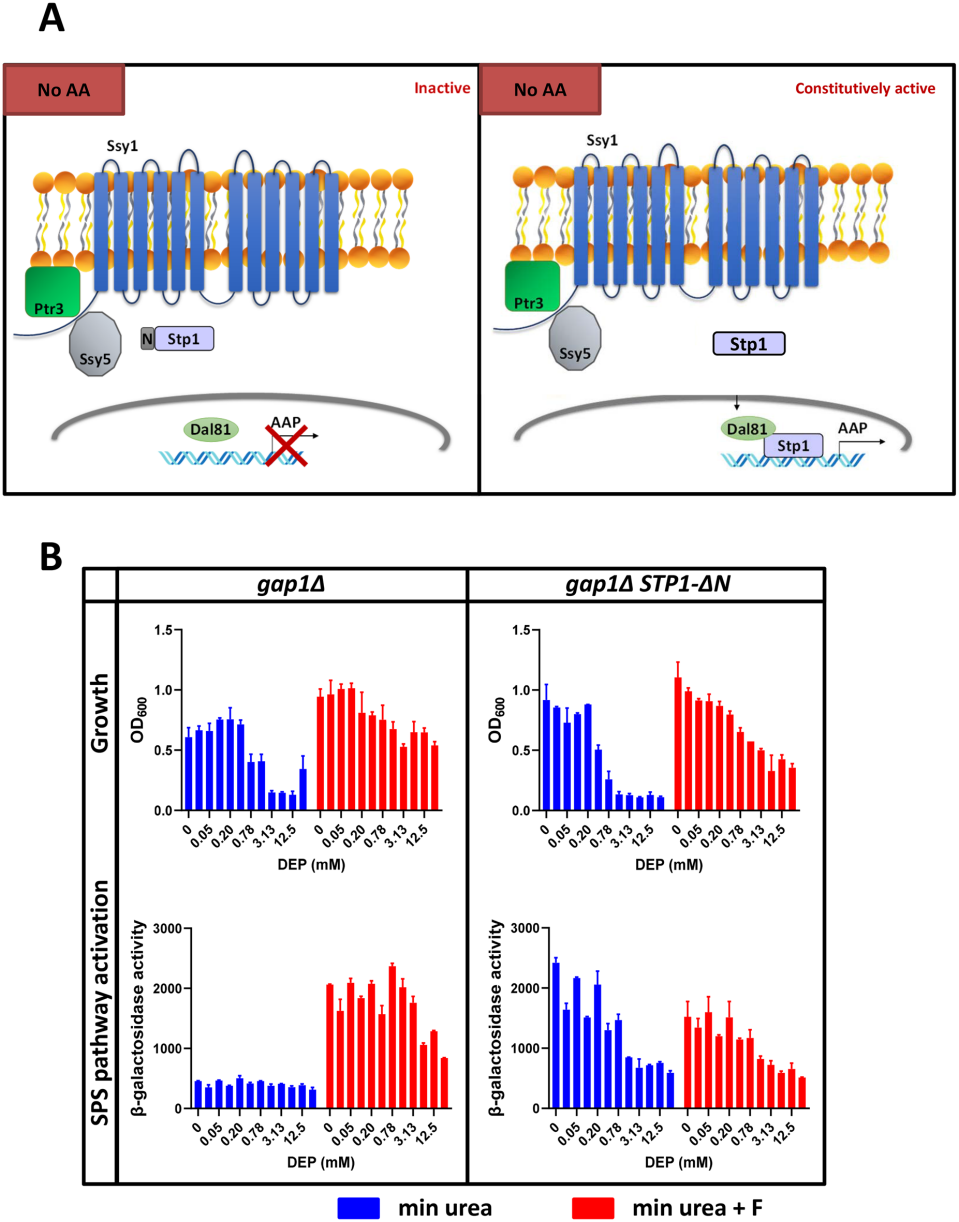
Constitutive activation of SPS pathway does not rescue DEP toxicity. (**A**) Schematic diagram comparing the inactive SPS pathway and constitutively activated SPS pathway in the absence of amino acids. (**B**) Constitutive activation of SPS pathway from Stp1ΔN in presence and absence of phenylalanine (5 mM). The reported values for OD_600_ and β-galactosidase activities in *gap1Δ* and *gap1Δ STP1ΔN* (Σ1278b background) are obtained from two independent experiments (mean ± SD). Two-fold serial dilution of DEP (maximum concentration at 25 mM) was used.

Activation of SPS signalling pathway results in the expression of amino acid permeases followed by transport of amino acids into the cytosol. We envisaged two possible mechanisms to explain how activation of SPS signalling pathway rescues DEP toxicity. Expression of amino acid permeases itself could rescue DEP toxicity. Alternatively, transport of amino acids to the cytosol by the permeases could rescue DEP toxicity. To distinguish between the two possibilities, we tested whether constitutive activation of the SPS-signalling pathway confers resistance to DEP. To constitutively activate SPS-signalling pathway, we deleted the N-terminal inhibitory domain of Stp1. Stp1ΔN can enter the nucleus and activate the permease gene expression even in the absence of extracellular amino acids^47^ (Fig. 6A). We either deleted the N-terminal domain of the genomic *STP1* copy or expressed truncated versions of Stp1 that constitutively activate the SPS pathway^47^. In both kinds of Stp1ΔN mutant strains, the SPS signalling pathway was activated even in the absence of extracellular amino acids (Figs. 6A and S4). However, constitutive activation of SPS-sensing pathway did not rescue DEP toxicity. DEP resistance was only observed in the presence of amino acids (Fig. 6B). These results indicate that the transport of extracellular amino acids to the cytosol by the amino acid permeases but not the expression of permeases per se, is required for conferring resistance to DEP.

### Amino acid catabolism is required for amino-acid mediated rescue of DEP toxicity

**Figure 7:**
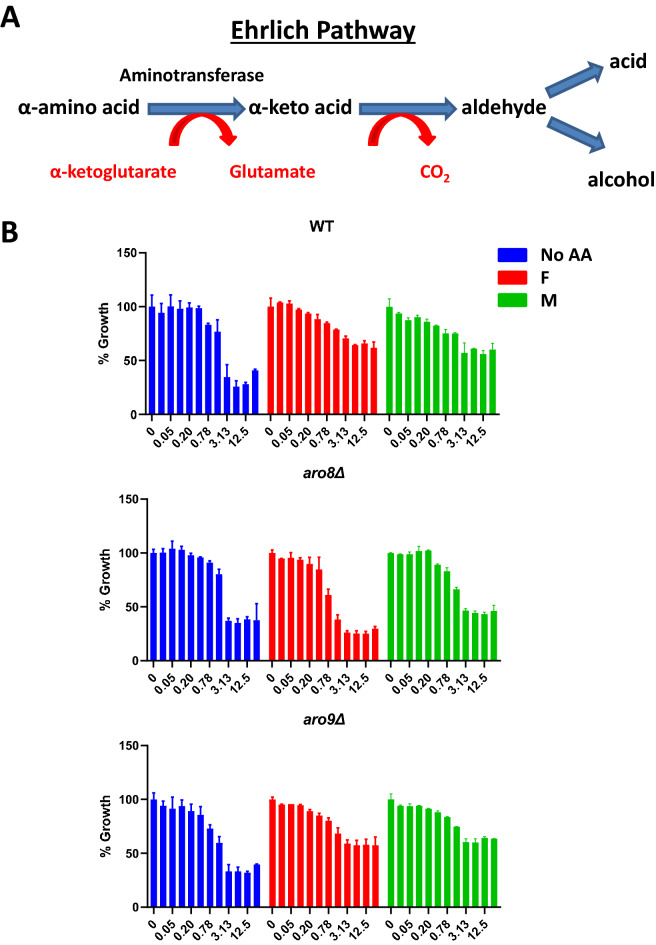
Catabolism of phenylalanine via the Ehrlich pathway is required for suppression of DEP-toxicity. (**A**) Catabolism of an α-amino acid via the Ehrlich pathway. (**B**) Wild type, *aro8Δ* and *aro9Δ* cells (Σ1278b background) growing in minimal urea medium in the absence (No AA) and presence of 5 mM phenylalanine (F)/methionine (M) were exposed to two-fold serial dilutions of DEP (maximum concentration at 25 mM). Normalized growth of various cultures is depicted as mean ± SD (*N* = 2).

Most amino acids are metabolized via the Ehrlich pathway to produce glutamate which contributes to about 85% of all nitrogen-containing compounds in yeast^48^ (Fig. 7A). We therefore tested whether degradation of amino acids via the Ehrlich pathway is required for amino acid rescue of DEP toxicity. For phenylalanine, the homologous aminotransferases I and II are Aro8 and Aro9, respectively are required for Ehrlich pathway^49^. We tested the ability of phenylalanine and methionine to rescue DEP toxicity in the wild type, *aro8*Δ and *aro9*Δ strains (Fig. 7B). Addition of amino acids to *aro9Δ* cells suppressed DEP toxicity. The rescue efficiency by phenylalanine and methionine in *aro9*Δ cells was 61% and 95.5% relative to rescue in wild type cells. Interestingly, rescue of DEP toxicity by phenylalanine and methionine was lost in *aro8*Δ cells. This is consistent with the report that Aro8 has broad specificity and can use methionine as a substrate^49^. Although *ARO8* and *ARO9* encode redundant enzymes, the genes are regulated differently^50^ which might account for the different phenotypes of *aro8Δ* and *aro9Δ* mutant cells. Our results strongly suggest that catabolism of amino acids via the Ehrlich pathway is required for amino-acid rescue of DEP toxicity.

### Structure–function studies of DEP’s effect on nitrogen metabolism

To identify the substructure of DEP that perturbs nitrogen metabolism in yeast, we tested the effect of a panel of compounds that bear structural similarity to DEP on the growth of yeast cells. We first compared the effects of diethyl phthalate, dimethyl isophthalic acid (DMIP), phthalic acid (PA) and isophthalic acid (IPA) on the growth of *gap1Δ* and *gap1Δ ssy1Δ* mutant cells in minimal urea medium either with or without phenylalanine. As seen for DEP, addition of amino acids rescued DMIP toxicity in *gap1Δ* cells but not in *gap1Δ ssy1Δ* cells (Fig. S5A). In contrast, PA and IPA had no effect on growth of yeast cells in minimal urea medium with/without phenylalanine (Fig. S5B).

As  PA is negatively charged, it might not be able to enter yeast cells to cause toxicity. On the other hand, DEP being uncharged could cross the plasma membrane and get hydrolysed to monoethyl phthalate (MEP) and PA by cytosolic esterases. We therefore investigated whether DEP is converted into PA in yeast cells. We incubated yeast cells with DEP and assayed the presence of PA and MEP in yeast cytosol by LC- mass spectrometry. However, neither MEP nor PA were detected by mass spectrometry in DEP-treated cells (Fig. S6), suggesting that DEP itself was the active inhibitor. We also found that the toxicity caused by aromatic monocarboxylic ester pentyl paraben was rescued by addition of amino acids in *gap1Δ* cells but not in *gap1Δ ssy1Δ* cells (Fig. S7). These results suggest that the aromatic monocarboxylic ester is the active chemical moiety that disrupts nitrogen metabolism in yeast. Consistent with this idea, esters of aromatic acid (DEP, DMIP and methyl benzoate) but not aliphatic carboxylic acids inhibited the growth of yeast cells (Fig. S8). Toxicity of aromatic esters was more pronounced in the *dal81Δ* and *stp1Δ* mutants in comparison to the wild type strain (Fig. S8).

### DEP alters the relative amino acid levels in yeast

**Figure 8:**
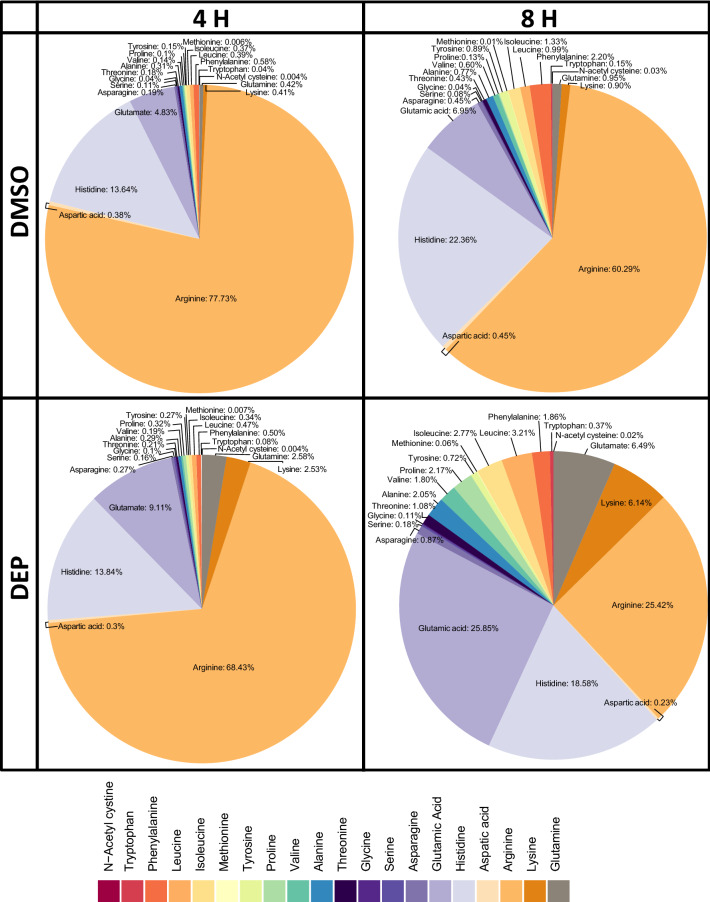
DEP alters the amino acid profile of yeast cells. Pie charts depicting the mean relative molar fractions of individual amino acids detected with mass spectrometry in ∑1278b wild type yeast cells treated with DMSO and 3.13 mM DEP in minimal urea medium for 4 and 8 h (*N* = 3).

Our results strongly suggest that DEP affects nitrogen metabolism in yeast cells. We used targeted metabolite analysis to test whether DEP affects the amino acid profile of yeast cells. We treated wild type yeast cells growing in minimal medium with either DMSO or DEP and then assayed the relative proportions of 20 amino acids after 4 and 8 h by mass spectrometry. We found that DEP had a major effect on the amino acid profile of yeast cells. Notably, there was an increase in the relative levels of glutamic acid and a decrease in the relative levels of arginine in DEP-treated cells in comparison to DMSO-treated cells (Figs. 8 and S9).

### DEP inhibits TORC1 activity in yeast cells

**Figure 9:**
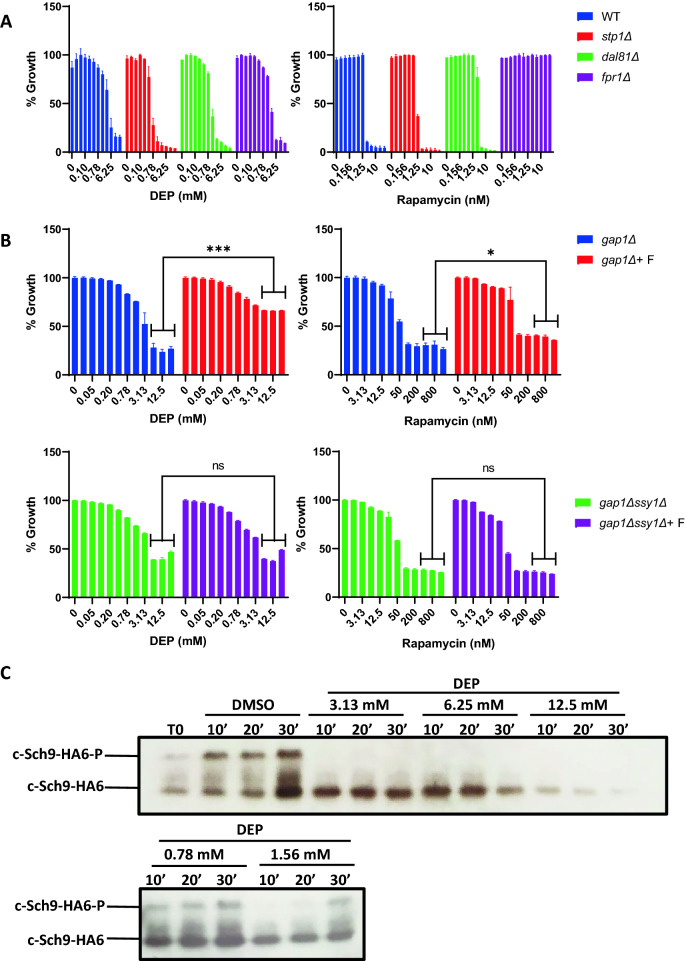
Treatment of yeast cells with DEP reduces TORC1 activity. (**A**) Comparison of normalized growth in wild type, *stp1Δ*, *dal81Δ* and *fpr1Δ* (S288C background) exposed to two-fold serial dilutions of DEP (maximal concentration at 25 mM) and Rapamycin (maximal concentration at 40 nM). (**B**) Normalized growth of *gap1Δ* and *gap1Δ ssy1Δ* cells (Σ1278b background) in minimal urea medium with and without phenylalanine (F) (5 mM) and exposed to two-fold serial dilutions of DEP (maximal concentration at 25 mM) and Rapamycin (maximal concentration at 1600 nM). Error bars represent mean ± SD from technical duplicates (One-tailed paired *t*-test, *P ≤ 0.05, **P < 0.01, ***P < 0.001). (**C**) Western blot of wild type yeast cells (Σ1278b background) treated with DMSO or DEP (0.78, 1.56, 3.13, 6.25 or 12.5 mM) for 10, 20 and 30 min. T0: Cycling cells. Unprocessed images of the two westerns are presented in Fig. S10).

Nitrogen and amino acid levels in yeast are coupled to growth and proliferation by the Target of Rapamycin Complex 1^51^. TORC1 is active in nutrient-replete medium but inactivated upon nutrient starvation^52^. Rapamycin inhibits TORC1 activity by forming a complex with *FPR1*-encoded prolyl isomerase FKBP12, which binds to TORC1’s catalytic subunit Tor1 and inhibits its kinase activity. To explore the interplay between DEP’s mode-of-action and TORC1 activity, we first compared the sensitivities of wild type, *stp1Δ*, *dal81Δ* and *fpr1Δ* cells to DEP and rapamycin. As seen previously, *stp1Δ* and *dal81Δ* cells were sensitive to DEP in comparison to wild type cells (Fig. 9A). As expected, *fpr1Δ* cells were resistant to rapamycin. Interestingly, *stp1Δ* cells were sensitive to rapamycin but *dal81Δ* cells were mildly resistant to rapamycin (Fig. 9A). Thus, mutations that increase DEP sensitivity in yeast either increase or decrease sensitivity to rapamycin. We then tested whether addition of amino acid to a poor nitrogen medium rescues the rapamycin sensitivity of *gap1Δ* and *gap1Δ ssy1Δ* cells. We used DEP as a control. As expected, addition of phenylalanine to the nitrogen-poor medium rescued the DEP-sensitivity in *gap1Δ* cells but not in *gap1Δ ssy1Δ* cells (Fig. 9B). Interestingly, addition of phenylalanine to the nitrogen-poor medium weakly rescued the rapamycin sensitivity of *gap1Δ* cells by 9.26% but not in *gap1Δ ssy1Δ* cells (Fig. 9B). Given that DEP and rapamycin cause similar growth phenotypes, we tested whether DEP affects TORC1 activity by examining the phosphorylation of its substrate Sch9. We grew yeast cells to log phase and then added either DMSO or DEP at varying concentrations (from 0.78 to 12.5 mM) to the culture and assayed Sch9 phosphorylation. The total Sch9 levels in cells treated with 12.5 mM DEP was reduced in comparison to untreated cells. However, treatment with lower concentrations of DEP (0.78–6.25 mM) caused a dose-dependent inhibition of Sch9 phosphorylation without affecting total Sch9 levels (Figs. 9C and S10). Thus, DEP treatment inhibits TORC1 activity in yeast. This inhibition could be either direct or indirect. As wild type and *fpr1*Δ cells are equally sensitive to DEP (Fig. 9A), FKBP12 is not required for DEP’s inhibitory effect on TORC1 activity. Precisely how DEP treatment causes TORC1 inhibition remains to be investigated.

In summary, our results show that DEP affects the growth of yeast cells by perturbing nitrogen metabolism.

## Discussion

DEP is a commonly used plasticizer that has toxic effects on human health. But the molecular mechanisms underlying its toxicity are poorly understood. Multiple lines of evidence indicate that DEP affects nitrogen metabolism in yeast. Firstly, two key transcriptional regulators of the amino-acid sensing pathway are sensitive to DEP. Secondly, toxicity of DEP was more pronounced in medium containing a poor nitrogen source which was rescued by addition of amino acids to the medium. Finally, DEP altered the amino acid profile of yeast cells and completely inhibited TORC1, a master regulator of Nitrogen/amino-acid signalling in yeast.

Based on our findings we propose a working model to explain how DEP affects growth of yeast cells and why addition of amino acids rescues DEP toxicity (Fig. S11). We hypothesize that DEP inhibits the growth of yeast cells by antagonizing TORC1 activity. It is well known that TORC1 activity is higher in nitrogen-rich medium compared to nitrogen-poor medium^51^ which would explain why DEP affects the growth of yeast cells much more severely in nitrogen-poor medium (urea) compared to in nitrogen-rich medium (ammonium sulphate). Addition of amino acids to the nitrogen-poor medium could promote TORC1 activity and overcome DEP inhibition. Consistent with this idea, addition of amino acids also rescued the rapamycin-sensitivity of yeast cells. Weaker rescue of rapamycin-sensitivity by amino acids in comparison to DEP-sensitivity, could be related to differences in their mechanisms of TORC1 inhibition. Glutamic acid produced by amino acid metabolism via the Ehrlich pathway could get converted into glutamine by the combined action of glutamate dehydrogenases and glutamine synthase. Glutamine thus produced could activate TORC1 and overcome DEP toxicity^53,54^. It is interesting to note that the relative levels of glutamic acid and glutamine increased with DEP treatment. Although increase in the relative glutamine levels might seem to contradict the model, rapamycin treatment of yeast cells also causes increase in the relative levels of glutamine and glutamate^55^. Inhibition of translation caused by TORC1 inactivation could alter the relative amino acid levels in the cell. Although our model (Fig. S11) is consistent with the existing data, it clearly requires further experimental validation. DEP could either directly inhibit TORC1 activity or indirectly by antagonising upstream activators of TORC1 or by affecting other cellular processes such as vacuolar function.

DEP was more toxic than its monoester variant MEP in both yeast and human cells. Intriguingly, the relative toxicity of another phthalate diester and monoester pair is exactly the opposite. Mono(2-ethylhexyl) phthalate (MEHP) is more potent than its corresponding diester, di-(2-ethylhexyl)-phthalate (DEHP), in yeast and mammalian cells^25,26^. Our previous studies showed that MEHP affected plasma membrane integrity. The hydrophobic 2-ethylhexyl group in MEHP might facilitate its interaction with the membrane bilayer. However, the smaller ethyl group in MEP might weakly interact with membranes accounting for its non-toxic effect on yeast cells. Both phthalic and isophthalic acids had no effect on yeast growth. As phthalic and isophthalic acids are negatively charged, they might not be able to cross the plasma membrane accounting for their non-toxic effects. MEHP is also charged but its effect could be mainly at the membrane and may need to enter the yeast cytoplasm. Comparing the uptake rates of phthalates and their various derivatives using mass spectrometric techniques will help to test our hypotheses.

Could our results in yeast have any bearing on mechanisms driving phthalate-related health problems in humans? Exposure to phthalates during early developmental stages has been linked with metabolic perturbations in humans^56,57,58^. A recent study examined the effect of prenatal exposure of diisononyl phthalate (DINP) on liver in mice. Intriguingly, Gene Ontology Enrichment analysis of differentially expressed genes in livers of exposed and control male mice revealed that ‘amino acid metabolic process’, ‘alpha-amino acid catabolic process’ and ‘cellular amino acid catabolic process’^59^ were among the top 5 GO terms. It would be interesting to investigate whether phthalates have any effect on amino-acid and nitrogen metabolism in human cells. Genome wide CRISPR KO screens in human cells^60^ can be used to interrogate the mechanism of diethyl phthalate toxicity in human cells and assess whether our observations in yeast are extendable to humans.

## Methods

### Strains and growth conditions

*S. cerevisiae* strains were used in this study along with their genotypes are presented in Table S1). Chemogenomic profiling and validation of DEP sensitivity experiments were performed with BY4743 strain and /or its derivatives. Yeast strains were grown in yeast extract-peptone-dextrose (YPD) medium and ammonia-based synthetic minimal dextrose (SD) medium at 30 °C. For experiments with the poor nitrogen source, the prototrophic ∑1278b strains were used, with urea or a single amino acid serving as the sole nitrogen source. ∑1278b cells were grown at 30 °C in Yeast Nitrogen Base without Amino Acids and Ammonium Sulfate medium (Difco, #233520) + 5 mM urea/amino acid + 3% glucose.

### Chemogenomic profiling

Chemogenomic profiling was performed using a pooled barcoded library derived from the *Saccharomyces cerevisiae* diploid homozygous deletion collection (Invitrogen) as described previously^25,26^. Pooled library of barcoded deletion strains was treated with either DMSO or 0.78 mM DEP. PCR-mediated amplification of barcode sequences, Next Generation Sequencing of the PCR products and analysis was done as previously described^25,26^.

### Gene ontology enrichment analysis

Gene Ontology Enrichment analysis of top hits (logFC < − 0.5 and *P*-value < 0.05) that confer resistance to DEP was performed using the web-based DAVID Gene Functional Classification Tool^36,37^. Gene ontology plots were visualised with REVIGO (http://revigo.irb.hr/)^38^.

### Construction of plasmids and yeast strains

PCR-based methods were used to delete genes in yeast. *AGP1p*-lacZ plasmid and Σ1278b strains were gifts from Prof. B. André (Physiologie Cellulaire, Université Libre de Bruxelles). Plasmids pCA022 (*STP1Δ131*), pCA023 (*STP1Δ132*), and pCA024 (*STP1Δ132*) encoding constitutively active alleles of *STP1*^47^ were gifts from Prof. Ljungdahl (Ludwig Institute for Cancer Research, Stockholm).

### Drug sensitivity assays

Drug sensitivity assays were performed using 200 μL cultures in 96-well culture plates (Corning, #3596) in two or three biological replicates. Serial dilutions (twofold) of diethyl phthalate (DEP) (Sigma-Aldrich, #524972), dimethyl phthalate (DMP) (Sigma-Aldrich, #525081), monoethyl phthalate (MEP) (TRC-M542580, #2306-33-4, Toronto Research Chemicals), dimethyl isophthalate (Sigma-Aldrich, #194239), isophthalic acid (Sigma-Aldrich, #I19209), ENA (Chembridge Corporation, #5123014), mono(2-ethylhexyl) phthalate (MEHP) (Wako Chemicals), methyl benzoate (Sigma-Aldrich, #M29908), ethyl hexanoate (Sigma-Aldrich, #148962), ethyl acetate (VWR Chemicals, #23882.321), ethyl propionate (Sigma-Aldrich, #112305), methyl hexanoate (Sigma-Aldrich, #W270806), ethyl butyrate (Sigma-Aldrich, #E15701), pentyl paraben (Sigma-Aldrich, #90744) and phthalic acid (Sigma-Aldrich, #80010) were performed with DMSO as a diluent. Initial inocula of OD_600_ measurements were determined to be 0.0625 for YPD HEPES and 0.2 for minimal media or Synthetic Complete (SC) media using the ND-1000 UV–visible light spectrophotometer (NanoDrop Technologies). Cell suspension was distributed in 96-well microtiter plate (200 μL/well) containing desired concentration of the compounds and incubated at 30 °C with shaking (220 rpm) overnight. All microplates were sealed with parafilm and incubated at 30 °C with shaking overnight. Optical density measurements were done with the microplate reader Gen 5TM (BIO-TEK Instrument, Vermont, USA).

### Testing the conversion of DEP into Phthalic acid

To evaluate whether DEP is being converted to MEP/PA by yeast cells, 100 mL yeast cell cultures (Σ1278b) were grown overnight in minimal urea medium with uracil at an initial OD_600_ of 0.2 in the presence of 3.13 mM DEP. Cells were pelleted the following day at 4500 rpm for 5 min. Medium was decanted and pellets were washed twice with cold PBS (25 mL) before freezing the pellets at − 80 °C before extraction. Cell pellets were extracted with 400 µL of ice-cold acetonitrile/methanol/water (ACN/MeOH/H_2_O, 2:2:1, v/v/v), sonicated in an ice-bath for 15 min, followed by centrifugation. The supernatant was transferred to a Liquid Chromatography vial with a glass insert for LC–MS analysis using an Agilent 1290 Infinity LC System coupled to an Agilent 6540 AccurateMass Q-TOF System. The extracts and reference standards were analysed on a reversed-phase C18 column using a linear gradient of water and acetonitrile with 0.1% formic acid as mobile phases and monitored in positive ionisation mode. DEP and its metabolites were identified by comparing the retention times of the reference standards.

### Measurement of relative amino acid levels by mass spectrometry

For the analysis for amino acid proportions, metabolite extraction and measurements were done as previously described^61,62^. Briefly, three biological replicates of yeast cell cultures (Σ1278b) were grown overnight in minimal urea medium with uracil and sub-cultured to an initial OD_600_ of 0.2 with 3.13 mM DEP or DMSO. Growth was monitored with OD measurements at time points 0, 2, 4 and 8 h following DEP treatment. 5 ml of each treated culture after 4 and 8 h were directly quenched in ice-cold methanol and centrifuged at 4500 rpm for 2 min at − 10 °C. Extraction solution kept at − 20 °C consisting of 40% (v/v) acetonitrile, 40% (v/v) methanol and 20% (v/v) water was used to resuspend and lyse the cells. This mixture was centrifuged at 4500 rpm for 5 min to remove cell debris. The supernatants were dried using vacuum evaporation and dried extracts were reconstituted in 100 µl of 98:2 water/methanol for liquid chromatography-mass spectrometry (LC–MS) analysis. LC–MS/MS analysis was conducted with Agilent 1290 ultrahigh pressure liquid chromatography system coupled to a 6490 Triple Quadrupole mass spectrometer fitted with a dual-spray electrospray ionization source with Jet Stream™ (Agilent Technologies, Santa Clara, CA). Chromatographic separation was obtained using an Acquity UPLC BEH Amide column (2.1 × 100 mm, 1.7 µm particle size) (Waters, Milford, USA). Setting for the oven temperature was fixed at 45 °C with a flow rate of 0.4 ml/min. The gradient elution utilised a mobile phase consisting of (A) water:acetonitrile (95:5, v/v) containing 10 mM ammonium formate and 0.1% formic acid and (B) water:acetonitrile (5:95, v/v) containing 10 mM ammonium formate and 0.1% formic acid. A 10 min linear decrease from 100 to 40% B was applied, which was held for 3 min and then returned to starting conditions over 0.1 min. The auto-sampler was cooled at 4 °C and 1 μL of injection volume was used. Electrospray ionization was conducted in positive ion mode with the following source parameters: drying gas temperature at 250 °C with a flow of 14 L/min, nebulizer gas pressure 40 psi, sheath gas temperature at 350 °C with a flow of 11 L/min, capillary voltage 4000 V and nozzle voltage 500 V. Multiple reaction monitoring (MRM) mode was applied to quantify amino acids and the collision energy for each MRM-transition was optimized by using a standard compound.

### Yeast β-galactosidase assay

Yeast cells (∑1278b) which express the *AGP1-lacZ* reporter gene were treated with DEP or DMSO and grown for 24–72 h in the presence or absence of amino acids. 200 μL of the cultures were transferred to 48-well plates and A_590_ measurements were taken. After yeast cells were lysed with 200 μL of lysis buffer (164 mM Na_2_HPO_4_, 24 mM NaH_2_PO_4_, 0.2% SDS) for 10 min in the incubator shaker. 400 μL of 1.13 mg/mL ortho-Nitrophenyl-β-galactoside (ONPG) (CAS #369-07-3) (Cayman Chemical, #26624), dissolved in Z buffer (60 mM Na_2_HPO_4_, 40 mM NaH_2_PO_4_, 10 mM KCl and 1 mM MgSO_4_ + 2-ME (2.7 μL of 2-Mercaptoethanol per mL of Z-buffer), was added to each well. Absorbance measurements at 414 nm (A_414_) were made after 15–30 min with the BioTek plate reader. To calculate beta-galactosidase activity, the following formula was used:

$$\frac{{1000 \times A_{414} }}{{OD_{590} \times Volume\left( {mL} \right) \times incubation\;time\left( {minutes} \right)}}$$

### Propidium iodide (PI) staining

PI staining was performed previously^25,26^. BY4743 cells (OD_600_≈1) were exposed to DEP (0.78, 1.25, 3.125, 6.25 mM), MEHP (2.25, 4.5, 9 mM) or DMSO in YPD for 30 min and 2 h. Cell pellets were washed twice with 1 × PBS, resuspended in 1 × PBS and treated with 5 μg/mL propidium iodide (PI) (Sigma-Aldrich, #P4170) in darkness for 20 min at 25 °C with shaking using a thermomixer. Evaluation of PI uptake by cells was done with bright-field and fluorescence microscopy (Excitation/Emission (nm): 535/617) and the percentage of PI-staining cells (N ≥ 100) was scored.

### TORC1 activity assay

Protein extraction, chemical fragmentation and western blot analysis was similar to that described by Urban et al.^63^. Overnight yeast culture (Σ1278b) was inoculated at an initial OD_600_ = 0.4 in SC media lacking uracil. After 5–6 h of incubation, the culture was split into the drug conditions DEP (0.78, 1.56, 3.13, 6.25, 12.5 mM) and DMSO. Aliquots of culture were collected per time point (0, 0.5, 1, and 2 h), mixed with tricholoroacetic acid (TCA) to a final concentration of 6% and kept on ice for at least 5 min. Afterwards, the cultures were centrifuged at 4500 rpm for 3 min and supernatants were decanted. Pellets were washed twice with ice-cold acetone and were then left to dry in a fume hood. Cell lysis and western blot procedures were subsequently carried out in the same manner as previously documented^64^.

### Statistical analysis

Data from sensitivity assays (*N* = 2) were analysed with one-tailed paired *t*-test between 2 groups (control and treated) to determine statistical significance using GraphPad Prism 9.0 software. Mean values were considered to be significant at *P* ≤ 0.0001(****), *P* ≤ 0.001(***), *P* ≤ 0.01(**) and *P* ≤ 0.05(*).

## Supplementary Information

Supplementary Information 1.

Supplementary Information 2.

## Data Availability

All datasets present in the current study are available from the corresponding author on reasonable request.

## Abbreviations

DEP: Diethyl phthalate
DEHP: Di-2-ethylhexyl phthalate
DMIP: Dimethyl isophthalate
MEP: Monoethyl phthalate
MEHP: Mono-2-ethylhexyl phthalate
PA: Phthalic acid
IPA: Isophthalic acid
TORC1: Target of rapamycin complex 1
