# Supplementary material for: Diethyl phthalate (DEP) perturbs nitrogen metabolism in Saccharomyces cerevisiae
Source: Sci Rep. 2022 Jun 17;12:10237. doi: 10.1038/s41598-022-14284-w (PMC9205984; doi:10.1038/s41598-022-14284-w)
Supplement: Supplementary file 1 — Supplementary Information 1. [file 41598_2022_14284_MOESM1_ESM.pdf]

Supplemental data

Legends for Supplemental Figures

Figure S1. DNA damage mutants are not sensitive to DEP

Comparison of normalized growth (OD_{600}) of individual HOP mutants (BY4743) treated with 1.56 mM DEP and 3.125 mM ENA. Error bars indicate standard deviation ($N = 2$).

Figure S2. The *stp1*Δ and *dal1*Δ mutants are sensitive to DEP but not to MEHP

Heat maps generated using GraphPad Prism 9.0, indicate normalized growth of wild-type (BY4743) and selected HOP mutants treated with MEHP and DEP ($N = 2$).

Figure S3. Toxicity caused by DEP but not MEHP is rescued by addition of amino acids to the medium

Normalized growth (OD_{600}) curves of overnight wild-type (Σ 1278b) cultures treated with a series of concentrations of DEP and MEHP in minimal Urea medium with 0, 2.5mM, 5mM or 10mM phenylalanine. Two-fold serial dilution of the compounds were used (maximum concentrations at 12.5 mM for MEHP, 25 mM for DEP).

Figure S4. Constitutive activation of SPS signalling pathway does not rescue DEP toxicity

Comparison of normalized growth (OD_{600}) between *gap1Δ* and *gap1Δ ssy1Δ* (Σ1278b background), which express the constitutively active *STP1 (*pCA022 or pCA023) or an empty vector (*pRS316*) against a series of DEP concentrations in the absence (-F) and presence (+F) of phenylalanine. Two-fold serial dilution of DEP (maximum concentration at 25 mM) was used. Error bars indicate standard deviation ($N = 2$).

Figure S5. PA and IPA are not toxic to yeast cells unlike their ester derivatives DEP and DMIP

(A) Comparison of normalized growth (OD_{600}) of wild-type (Σ1278b) and *gap1Δ ssy1Δ* treated with DEP or DMIP after 24 h. Two-fold serial dilution of the compounds were used (maximum concentrations at 25 mM for DEP, 6.25 mM for DMIP).

(B) Comparison of normalized growth (OD_{600}) of wild-type (Σ1278b) and *gap1Δ ssy1Δ* treated with PA or IPA after 24 h. Two-fold serial dilution of the compounds were used (maximum concentrations at 25 mM for PA and 6.25 mM for IPA). Error bars indicate standard deviation ($N = 2$).

Chemical structures illustrating the respective compounds are indicated on the left of each graph.

Figure S6. DEP is not converted to PA in yeast cells

LC-MS profiles of yeast cells treated with 3.13 mM DEP (left) and standards (right). Top: TIC of yeast cell extract; (a) EIC of m/z 223.09703 (DEP); (b) EIC of m/z 195.06573 (MEP); (c) EIC of m/z 167.03443 (PA, not detected in yeast cell extract).

Figure S7. Amino acid addition rescues both DEP and Pentyl paraben toxicity

Comparison of growth percentages in wild type (Σ1278b) cells treated with DEP (1.56 mM) or Pentyl paraben (0.195 mM) in the absence (No AA) and presence of amino acids phenylalanine (F) and Leucine (L). Error bars indicate standard deviation ($N = 2$) (One-tailed paired *t*-test: * implies $P < 0.05$).

Figure S8. Aromatic esters but not aliphatic esters are toxic to yeast cells

Comparison of growth (OD_{600}) with aromatic versus aliphatic esters. Wild type, *stp1Δ, dal81Δ* and *mms1Δ* cells were treated with a series of concentrations for the compounds DEP, methyl benzoate, DMP, ethyl hexanoate, ethyl acetate, ethyl propionate, methyl hexanoate and ethyl butyrate. Two-fold serial dilution of the compounds were used (maximum concentrations at 25 mM for DEP, ethyl acetate, ethyl propionate and ethyl butyrate, 12.5 mM for methyl benzoate, DMIP, ethyl hexanoate and methyl hexanoate). Error bars indicate standard deviation (*N* = 2).

Figure S9. DEP alters the amino acid profile of yeast cells

Relative molar percentages of various amino acids in DMSO- and DEP-treated cultures after 4 h and 8 h of incubation in minimal Urea medium as detected by mass-spectrometry. Error bars indicate standard deviation (*N* = 3).

Figure S10 Treatment of yeast cells with DEP reduces TORC1 activity

A) Unprocessed image of two western blots related to Figure 9C is presented here.

B) Annotation of samples in the processed image derived from the two western blots in A. Please note that the western blot at the top includes protein samples from DMSO-treated yeast cells (duplicated in the bottom western blot) and positive-control cells treated with 40 nM rapamycin (this was not essential as DEP inhibited TORC1), which were not included in the images presented in Figure 9C.

Figure S11. Working model for DEP's mechanism of growth inhibition of yeast cells

A) TORC1 activity is low in poor nitrogen medium. DEP further inhibits TORC1 activity and disrupts nitrogen metabolism and growth.

B) In the presence of amino acids, SPS pathway is activated, which results in amino acid uptake. Catabolism of amino acids produces glutamate (Glu) via the Ehrlich pathway.

Glutamate gets converted to glutamine (Gln) which then activates TORC1 in overcoming the inhibition caused by DEP.

Table S1. Yeast strains used in the study

**Strain number**	**Genotype**	**Reference**
4847	MATα *ura3 lys2 ho::LYS2*	Iraqui *et al.* (1999)
4848	MATa *gap1Δ::kanMX2 ura3 lys2 ho::LYS2*	Iraqui *et al.* (1999)
4849	MATα *gap1:: KanMX2 stp1::KanMX2 stp2::kanMX2 dal81:KanMX2 ura3 lys2 ho::LYS2*	Abdel-Sater (2004)
4863	MAT*a gap1*Δ::*kanMX2 ssy1*Δ::*NatMX6 ura3 lys2 ho::LYS2*	This study
4864	MATα *ssy1*Δ:*NatMX4 ura3 lys2 ho::LYS2*	This study
CG1	MATα *ura3 lys2 ho::LYS2 pAGP1-lacZ::URA3*	This study
CG2	MATα *gap1Δ:KanMX2 ssy1Δ:kanMX2 ura3 pAGP1-lacZ::URA3*	This study

CG3/4894	MATα *gap1Δ:kanMX2 ura3 pAGP1-lacZ::URA3*	This study
CG4	MATα *gap1Δ::KanMX2 stp1Δ:KanMX2 stp2Δ:KanMX2 ura3 pAGP1-lacZ::URA3*	This study
CG9	MATa *gap1*Δ::*kanMX2 ssy1*Δ::*NatMX6ura3 lys2 ho::LYS2* pCA022 -*STP1Δ131::URA3*	This study
CG10	MATa *gap1*Δ::*kanMX2 ssy1*Δ::*NatMX2 ura3 lys2 ho::LYS2* pCA022-*STP1Δ132::URA3*	This study
CG12	MATa *gap1*Δ::*kanMX2 ssy1*Δ::*NatMX2 ura3 lys2 ho::LYS2*	This study
CG13	MATa *gap1Δ::kanMX2 ura3 lys2 ho::LYS2* pCA022 -*STP1Δ131*	This study
CG14	MATa *gap1Δ::kanMX2 ura3 lys2 ho::LYS2* pCA022 -*STP1Δ132*	This study
CG16	MATa *gap1Δ::kanMX2 ura3 pRS316*	This study
CG17	MATa *gap1Δssy1Δ stp1ΔN*	This study

Table S2. Homozygous profiling data for DEP.

Excel worksheet ‘DEP’ contains the raw HOP data indicating the logFC values for 4817 deletion strains along with the corresponding P-value, logCPM (Counts per Million), Likelihood Ratio (LR) and False Discovery Rate (FDR).

Excel worksheet ‘logFC<-0.5 PVALUE<0.05,’ contains the list of 78 genes that confer resistance to DEP.

Excel Worksheet ‘GO’ (Gene Ontology) contains enrichment analysis results for 78 genes obtained using DAVID for the GO categories namely, Biological Process (BP), Cellular Component (CC) and Molecular Function (MF).

Excel Worksheet ‘REVIGO’ contain the REVIGO Tree analysis of the overrepresented GO terms obtained with DAVID (GO terms with a P-value<0.05).

#

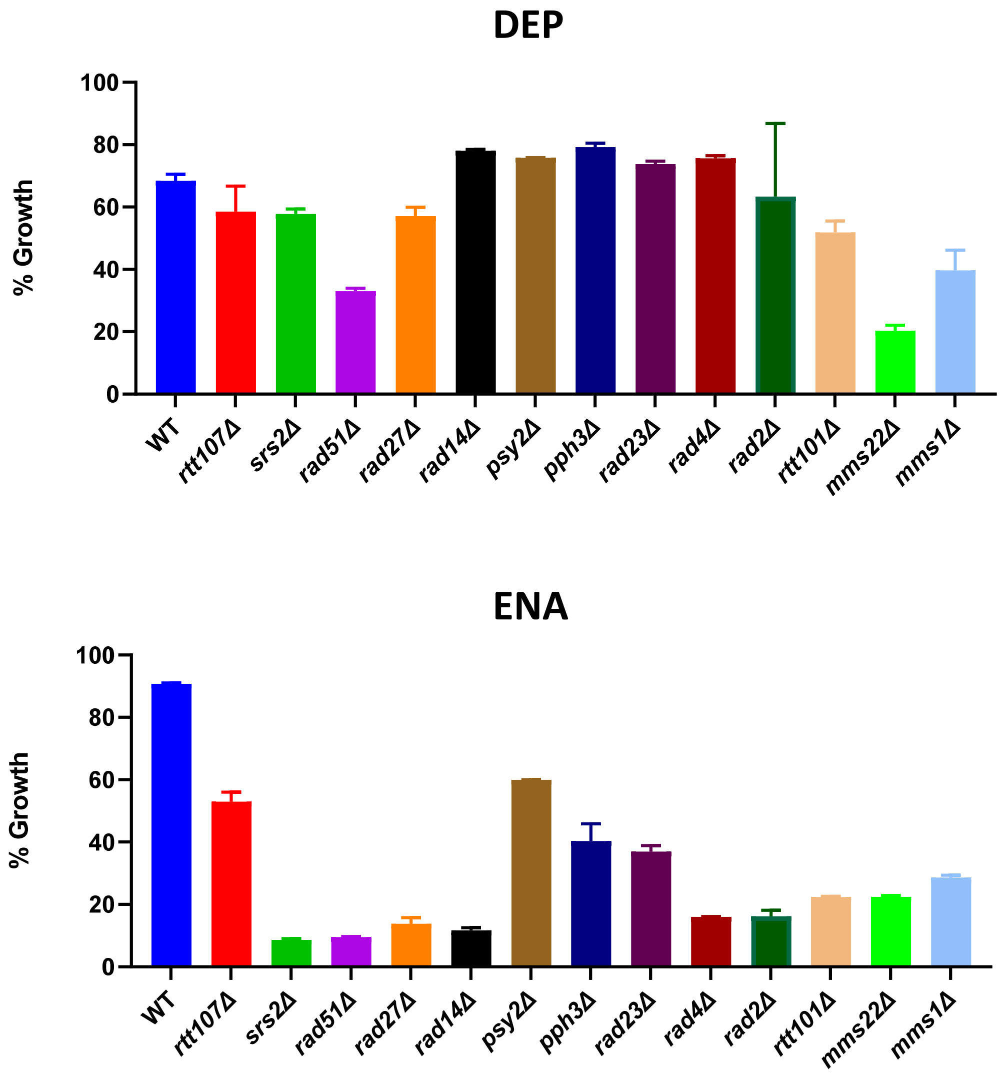
DEP
% Growth
100
80
60
40
20
0
WT
rtt107Δ
srs2Δ
rad51Δ
rad27Δ
rad14Δ
psy2Δ
pph3Δ
rad23Δ
rad4Δ
rad2Δ
rtt101Δ
mms22Δ
mms1Δ
ENA
% Growth
100
80
60
40
20
0
WT
rtt107Δ
srs2Δ
rad51Δ
rad27Δ
rad14Δ
psy2Δ
pph3Δ
rad23Δ
rad4Δ
rad2Δ
rtt101Δ
mms22Δ
mms1Δ

Figure S1

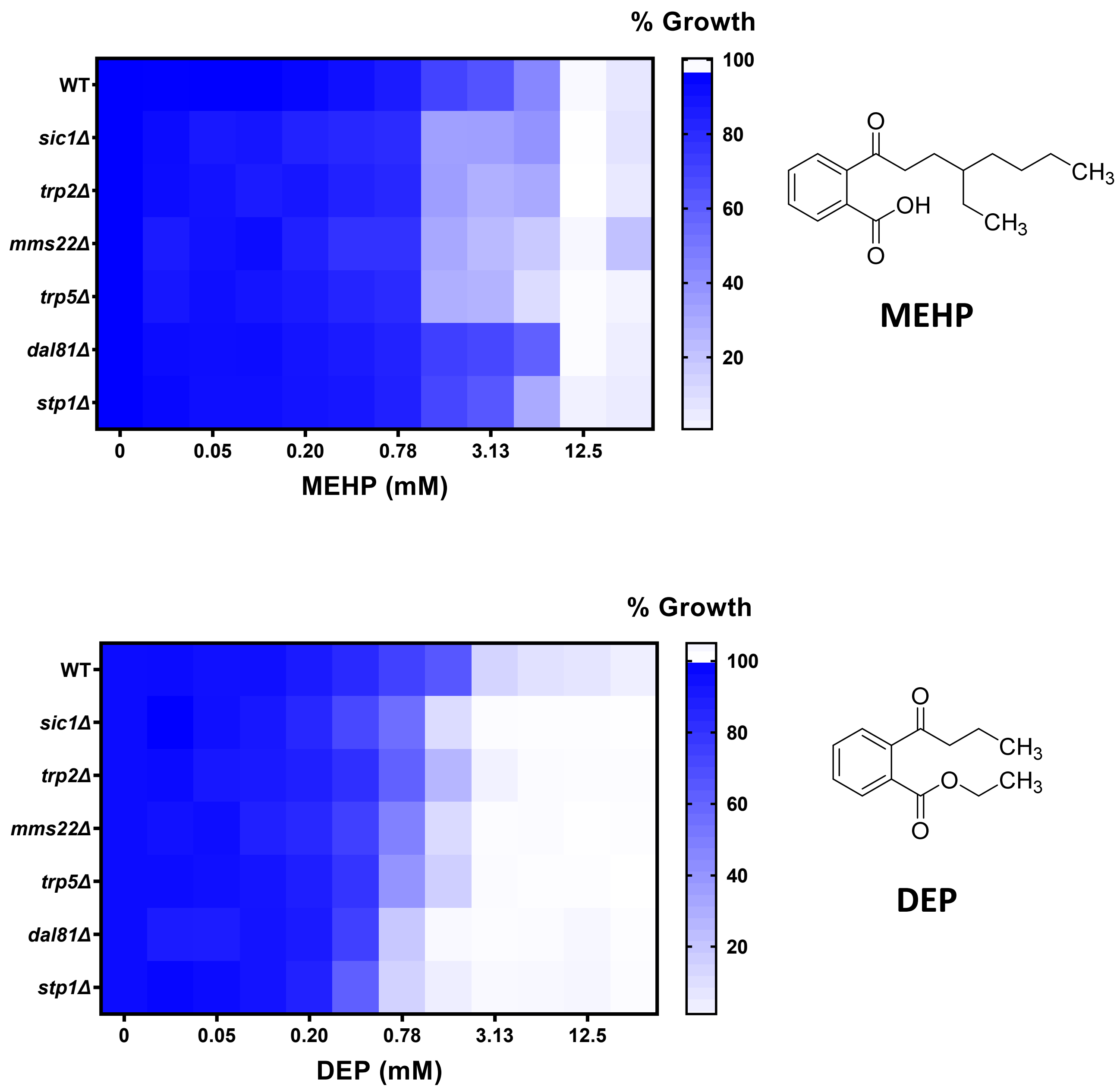
% Growth
100
80
60
40
20
WT
sic1Δ
trp2Δ
mms22Δ
trp5Δ
dal81Δ
stp1Δ
0
0.05
0.20
0.78
3.13
12.5
MEHP (mM)
O
OH
O
CH3
CH3
MEHP
% Growth
100
80
60
40
20
WT
sic1Δ
trp2Δ
mms22Δ
trp5Δ
dal81Δ
stp1Δ
0
0.05
0.20
0.78
3.13
12.5
DEP (mM)
O
CH3
O
CH3
O
DEP

Figure S2

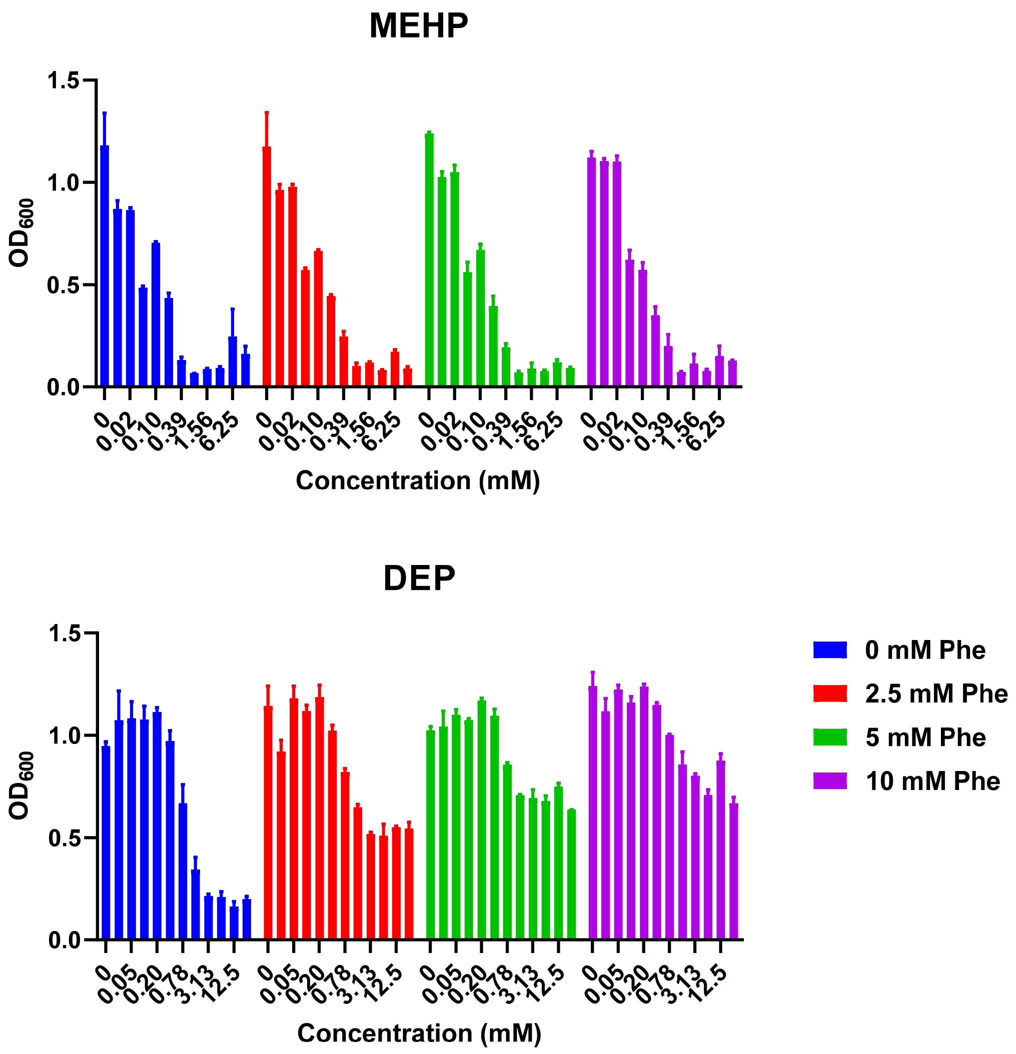
MEHP
1.5
1.0
0.5
0.0
OD600
0
0.02
0.10
0.39
1.56
6.25
Concentration (mM)
DEP
0
0.05
0.20
0.78
3.13
12.5
0 mM Phe
2.5 mM Phe
5 mM Phe
10 mM Phe

Figure S3

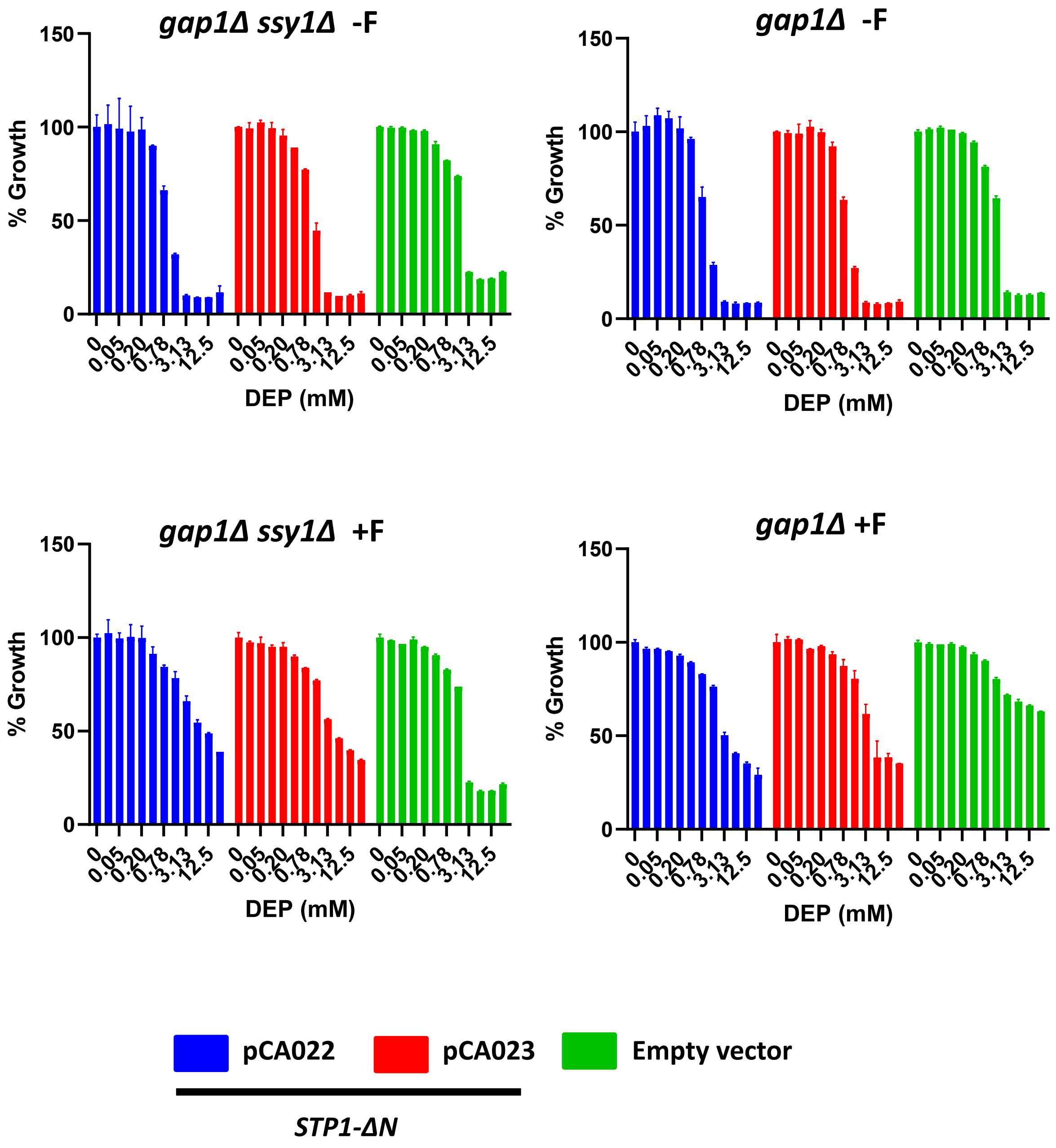
gap1Δ ssy1Δ -F
gap1Δ -F
gap1Δ ssy1Δ +F
gap1Δ +F
% Growth
150
100
50
0
0
0.05
0.20
0.78
3.13
12.5
DEP (mM)
pCA022
pCA023
Empty vector
STP1-ΔN

Figure S4

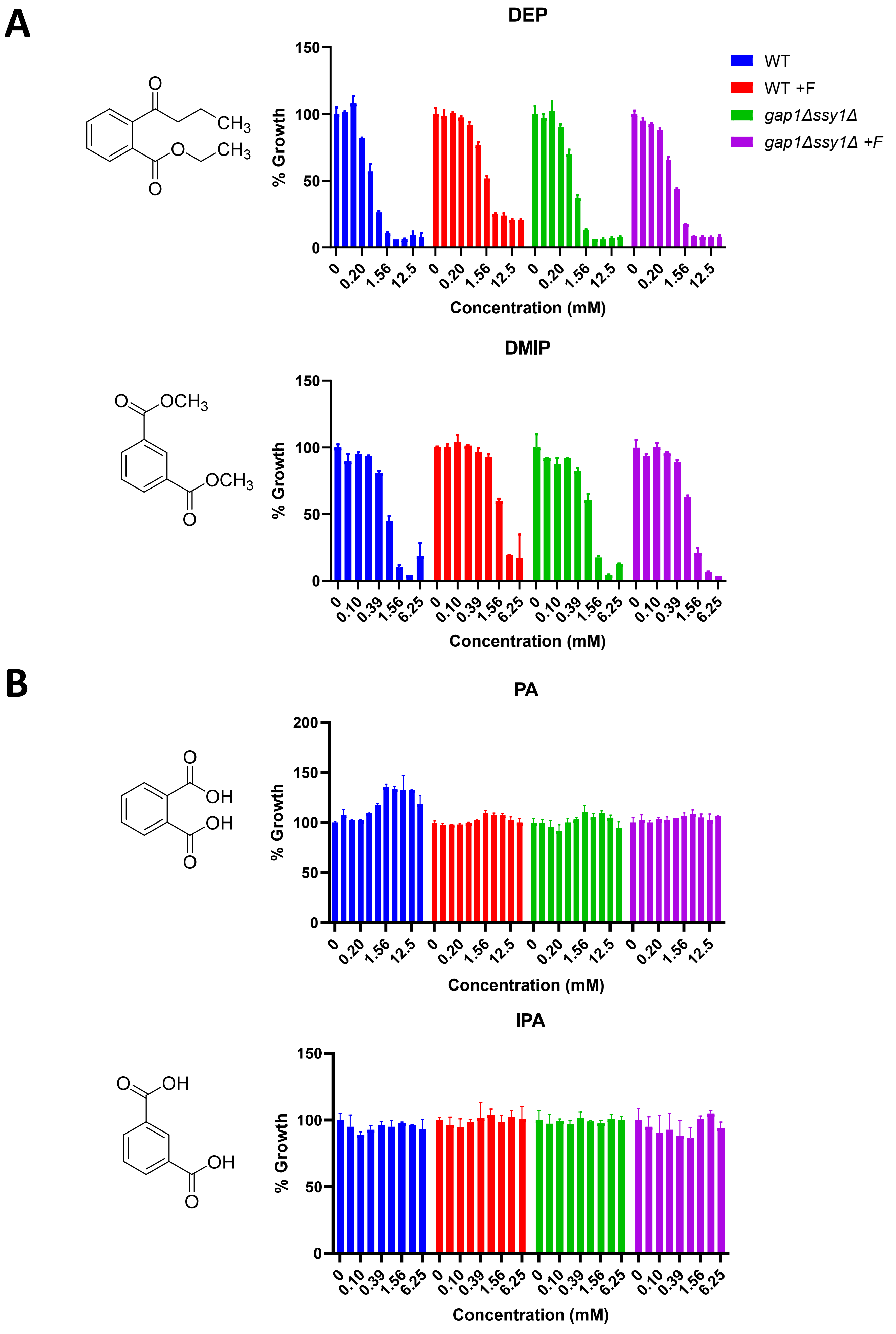
A
DEP
DMIP
B
PA
IPA
O
CH3
O
CH3
O
OCH3
OCH3
OH
WT
WT +F
gap1Δssy1Δ
gap1Δssy1Δ +F
% Growth
Concentration (mM)
0
0.20
1.56
12.5
0.10
0.39
6.25
50
100
150
200

Figure S5

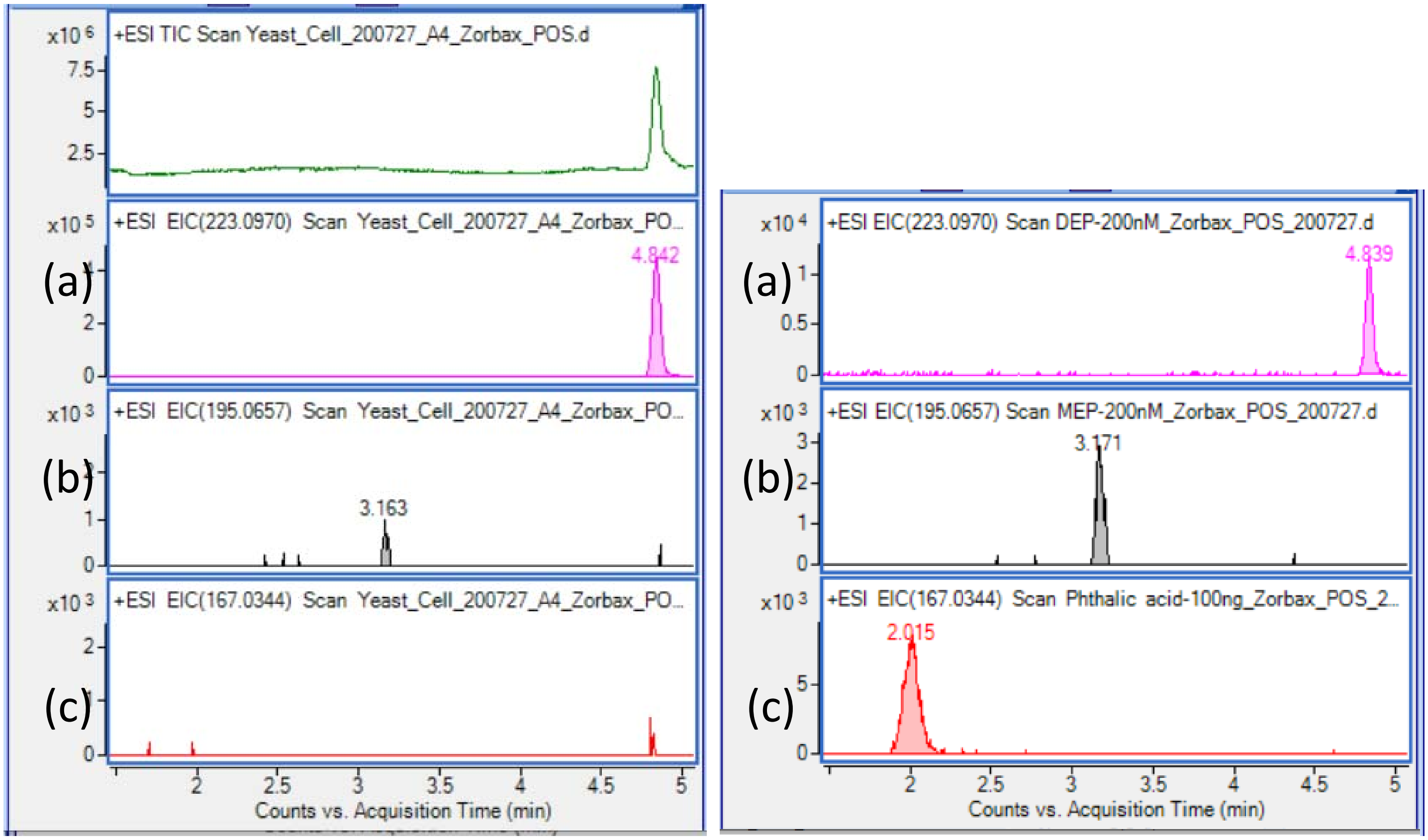

+ESI TIC Scan Yeast_Cell_200727_A4_Zorbax_POS.d
x10 6
7.5
5
2.5
+ESI EIC(223.0970) Scan Yeast_Cell_200727_A4_Zorbax_PO...
x10 5
(a)
4.842
2
0
+ESI EIC(195.0657) Scan Yeast_Cell_200727_A4_Zorbax_PO...
x10 3
(b)
3.163
1
0
+ESI EIC(167.0344) Scan Yeast_Cell_200727_A4_Zorbax_PO...
x10 3
2
(c)
0
2
2.5
3
3.5
4
4.5
5
Counts vs. Acquisition Time (min)
+ESI EIC(223.0970) Scan DEP-200nM_Zorbax_POS_200727.d
x10 4
(a)
4.839
1
0.5
0
+ESI EIC(195.0657) Scan MEP-200nM_Zorbax_POS_200727.d
x10 3
(b)
3.171
3
2
1
0
+ESI EIC(167.0344) Scan Phthalic acid-100ng_Zorbax_POS_2...
x10 3
(c)
2.015
5
0
2
2.5
3
3.5
4
4.5
5
Counts vs. Acquisition Time (min)

Figure S6

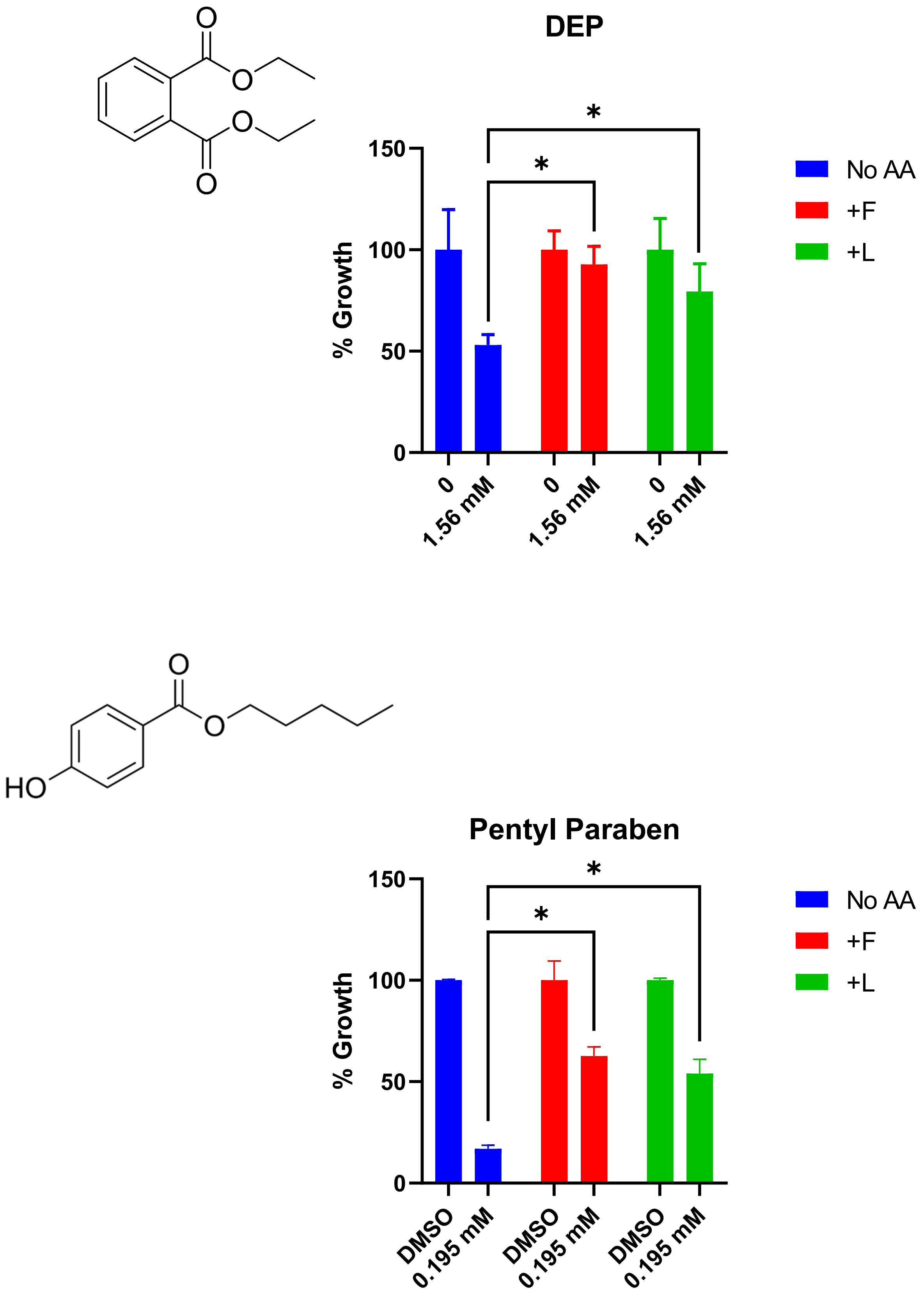

O
O
O
O
DEP
*
*
150
100
50
0
% Growth
0
1.56 mM
0
1.56 mM
0
1.56 mM
No AA
+F
+L
O
O
HO
Pentyl Paraben
*
*
150
100
50
0
% Growth
DMSO
0.195 mM
DMSO
0.195 mM
DMSO
0.195 mM
No AA
+F
+L

Figure S7

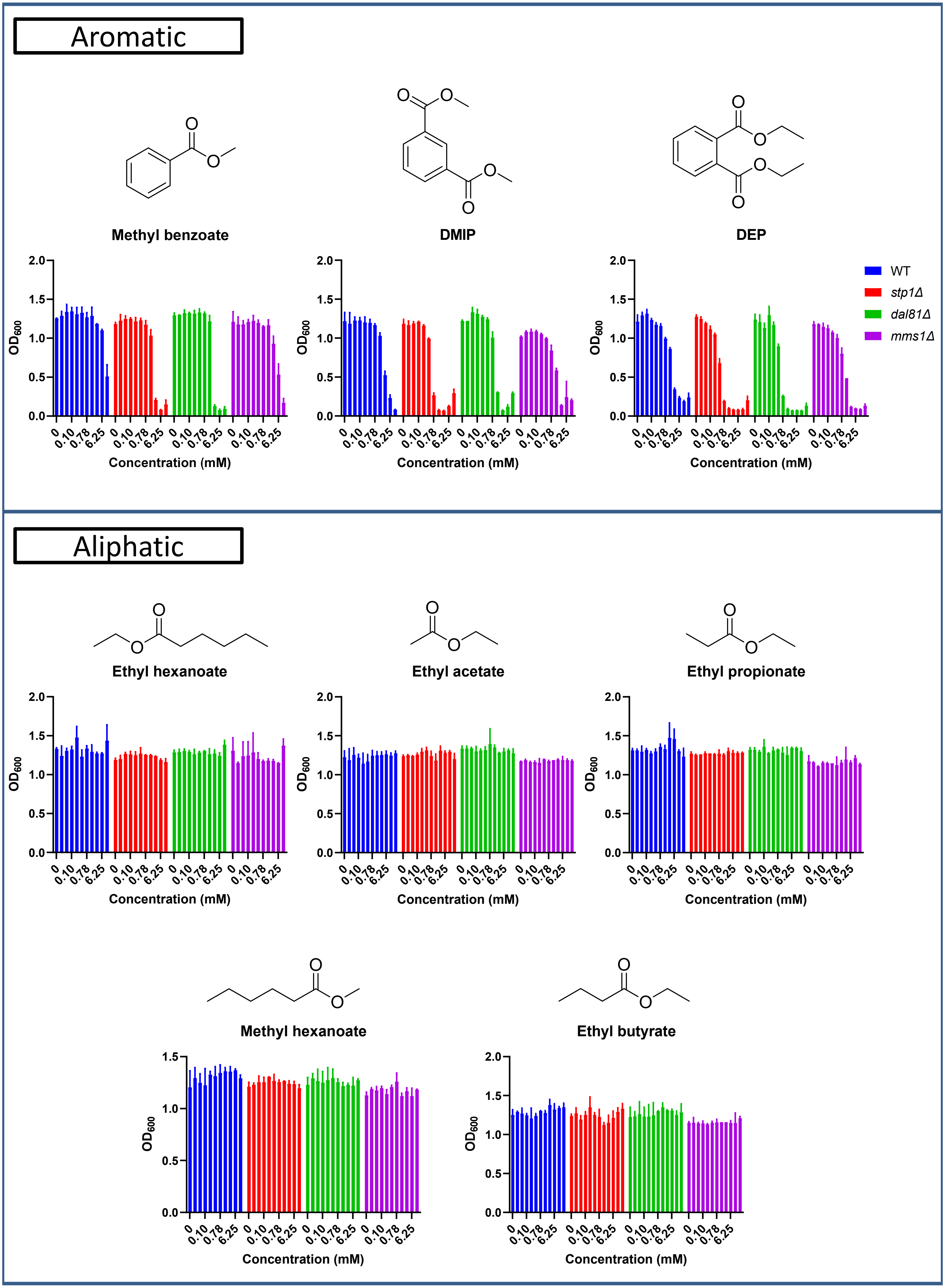
Aromatic
Methyl benzoate
DMIP
DEP
WT
stp1Δ
dal81Δ
mms1Δ
OD600
Concentration (mM)
0
0.10
0.78
6.25
Aliphatic
Ethyl hexanoate
Ethyl acetate
Ethyl propionate
Methyl hexanoate
Ethyl butyrate

Figure S8

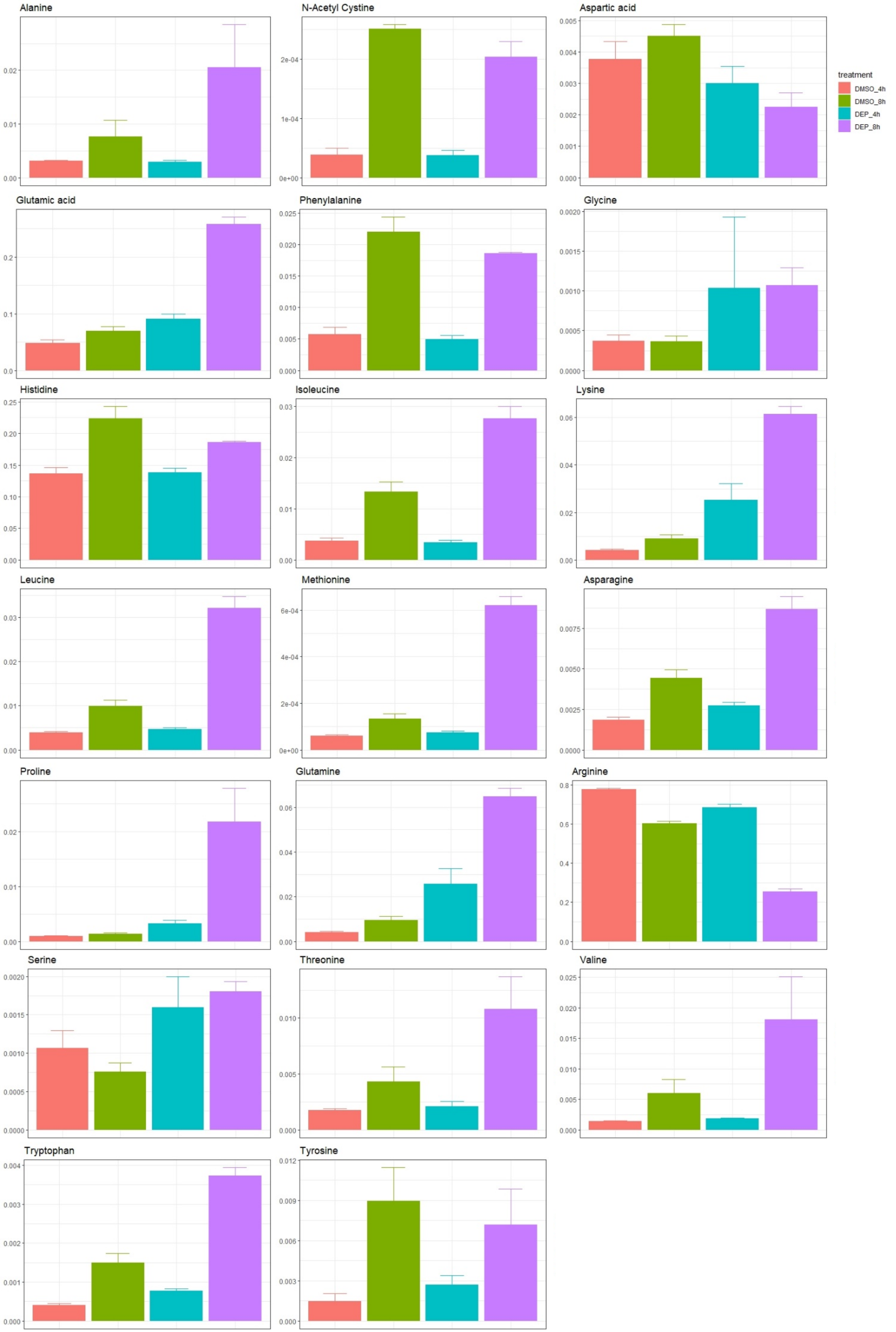
Alanine
N-Acetyl Cystine
Aspartic acid
treatment
DMSO_4h
DMSO_8h
DEP_4h
DEP_8h
Glutamic acid
Phenylalanine
Glycine
Histidine
Isoleucine
Lysine
Leucine
Methionine
Asparagine
Proline
Glutamine
Arginine
Serine
Threonine
Valine
Tryptophan
Tyrosine

Figure S9

A

Poor Nitrogen Medium + DEP

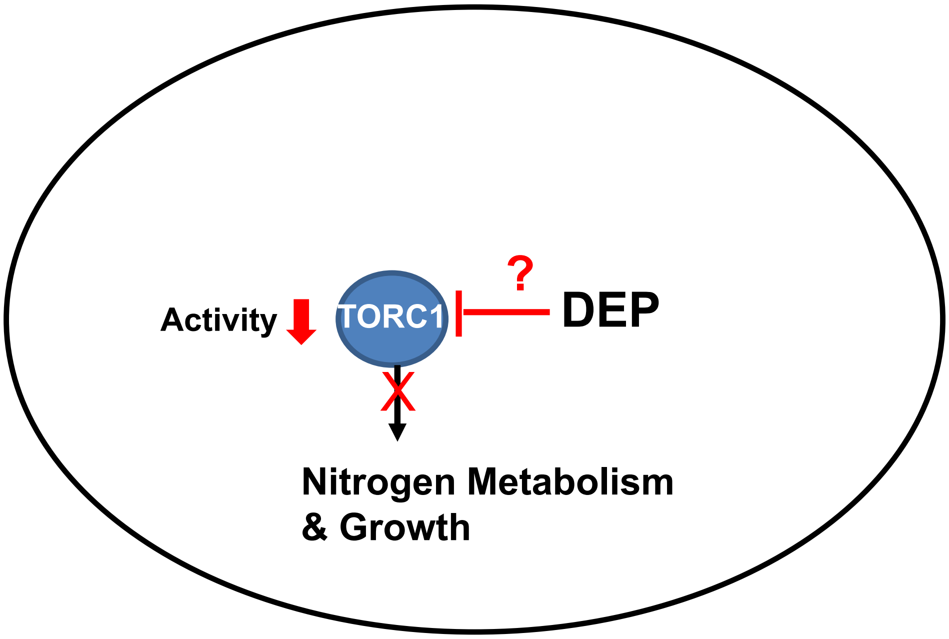

B

Poor Nitrogen Medium + DEP + Amino acids (AA)

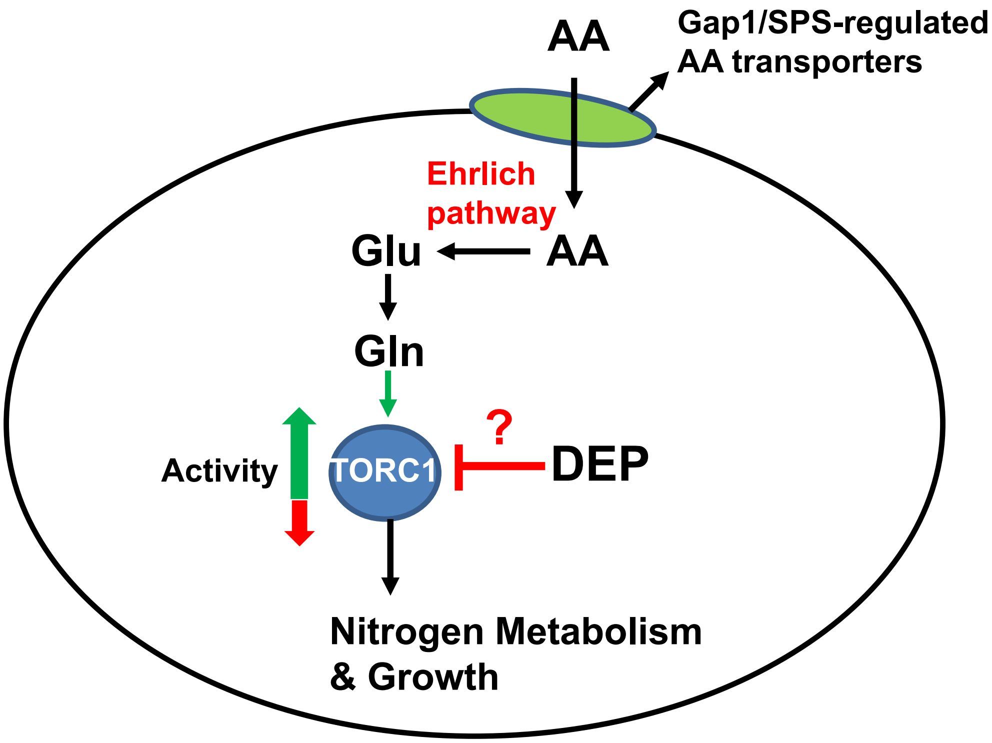

Figure S10
